# Saturation Mutagenesis of the HIV-1 Envelope CD4 Binding Loop Reveals Residues Controlling Distinct Trimer Conformations

**DOI:** 10.1371/journal.ppat.1005988

**Published:** 2016-11-07

**Authors:** Maria Duenas-Decamp, Li Jiang, Daniel Bolon, Paul R. Clapham

**Affiliations:** 1 Program in Molecular Medicine, Biotech 2, University of Massachusetts Medical School, Worcester, United States of America; 2 Biochemistry and Molecular Pharmacology, Lazare Research Building, University of Massachusetts Medical School, Worcester, United States of America; Miller School of Medicine, UNITED STATES

## Abstract

The conformation of HIV-1 envelope (Env) glycoprotein trimers is key in ensuring protection against waves of neutralizing antibodies generated during infection, while maintaining sufficient exposure of the CD4 binding site (CD4bs) for viral entry. The CD4 binding loop on Env is an early contact site for CD4 while penetration of a proximal cavity by CD4 triggers Env conformational changes for entry. The role of residues in the CD4 binding loop in regulating the conformation of the trimer and trimer association domain (TAD) was investigated using a novel saturation mutagenesis approach. Single mutations identified, resulted in distinct trimer conformations affecting CD4bs exposure, the glycan shield and the TAD across diverse HIV-1 clades. Importantly, mutations that improve access to the CD4bs without exposing the immunodominant V3 loop were identified. The different trimer conformations identified will affect the specificity and breadth of nabs elicited in vivo and are important to consider in design of Env immunogens for vaccines.

## Author Summary

Spike proteins on the surface of HIV virus particles bind to CD4 receptors on the surface of immune cells and trigger infection. The immune system in an infected person attacks the virus spikes by producing antibodies that bind and neutralize them. To combat this immune attack, HIV continually alters the structure of the spike and thus escapes host antibodies. However, this process must still preserve sites on the spike that bind CD4 receptors for infection. Here, we investigated how the spike regulates its structure. We used a systematic approach to investigate every possible mutation covering a region of the spike critical for binding the CD4 receptor and controlling overall structure. We identified different sites and mechanisms that control the spike structure for diverse HIV-1 strains and impact the exposure of the binding site for CD4 along with targets for neutralizing antibodies. Our observations will help guide the design of spike structures for vaccines that induce neutralizing antibodies effective against different HIV-1 strains across the globe.

## Introduction

The HIV-1 envelope glycoprotein (Env) comprises a surface gp120 and a transmembrane gp41 non-covalently associated on heterodimeric trimers. When gp120 on the Env trimer binds CD4 at the cell surface, conformational changes are triggered that open the trimer to expose a site for binding to a coreceptor, usually CCR5. Trimer opening involves the disengagement of the trimer association domain (TAD) at the trimer apex enabling (1) movement of the V1V2 loops to expose the V3 loop and (2) full exposure of determinants on the V1V2 stem recruited by CD4 to assemble the bridging sheet. The V3 loop and sections of the bridging sheet form the coreceptor binding site [1].

**Fig 1:**
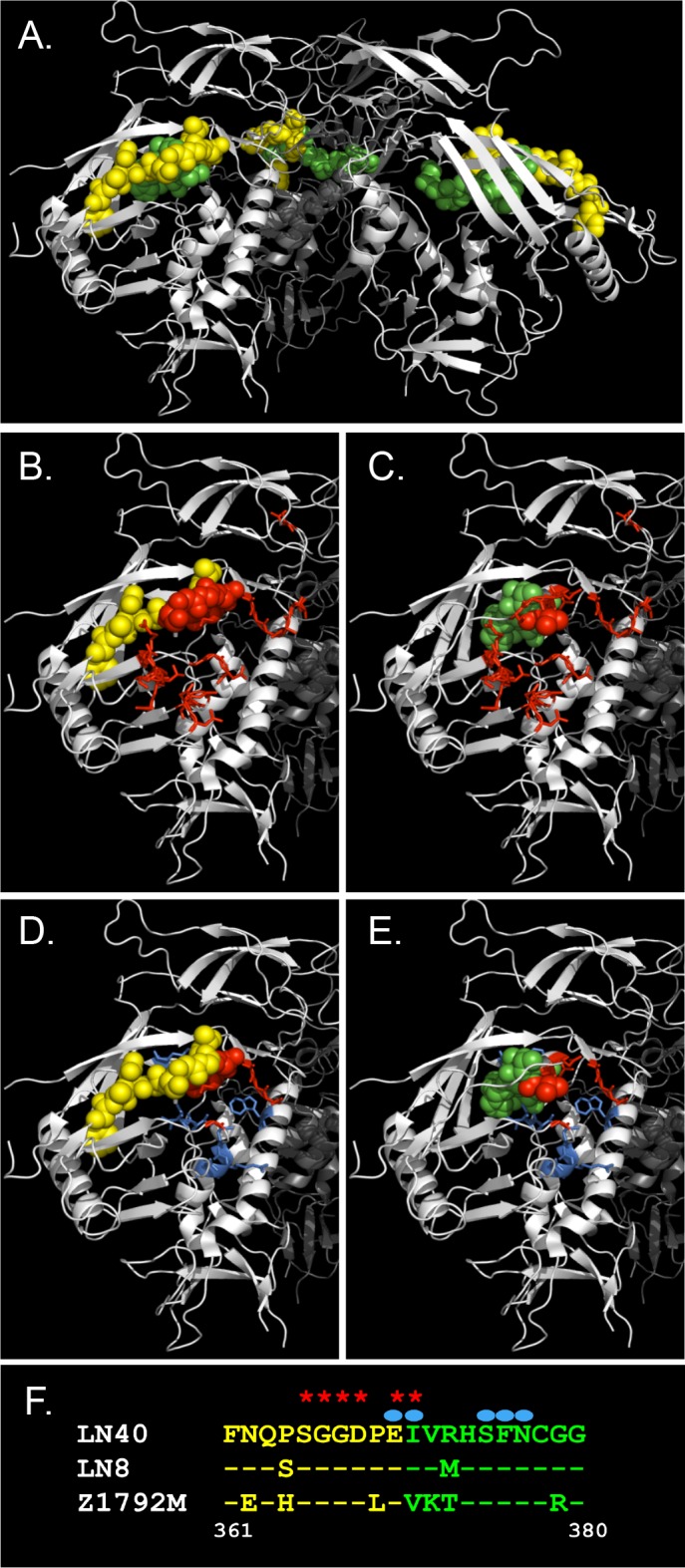
The HIV-1 envelope trimer structure. Structures shown were derived from PDB 4NCO [12]. (A) Side view of trimer depicted as a cartoon showing the location of the target sequences of two EMPIRIC libraries that span the CD4 binding loop and flanks and comprise residues 361–370 (yellow spheres) and 371–380 (green spheres). (B,C,D,E) Residues 361–370 (B,D) and 371–380 (C,E) are shown as spheres. (B-C) Red amino acids indicate CD4 contact residues. (D-E) Both blue and red spheres surround the Phe-43 cavity, with the red spheres indicating those that directly contact Phe-43 [2]. (F) CD4 binding loop residues 361–380 for clade B Envs, LN40, LN8 and clade C Env Z1792M. Stars denote CD4 contact residues, circles denote residues that line the Phe-43 cavity.

The CD4 binding loop on Env is an early contact site for CD4 [2], while penetration of a proximal cavity by the hydrophobic side chain of CD4’s Phe-43 triggers Env conformational changes and trimer opening [3–5] (Fig 1). For example, an S375W substitution in Env results in the organic side chain of the tryptophan accessing the cavity and directing a more open Env conformation [4, 5].

HIV-1 Envs in brain tissue use CCR5 as a coreceptor and are highly macrophage-tropic. These Env variants interact efficiently with low CD4 levels on macrophages for infection [6, 7]. Determinants that modulate mac-tropism of R5 Envs lie within or proximal to the CD4bs [8, 9] as well as in V1V2 and V3 loops of the TAD [8, 10, 11]. They include residues within the variable N-terminal flank of the CD4 binding loop that together with V3 loop amino acids modulated mac-tropism in a highly mac-tropic brain Env from a subject with neurological complications [8].

Here, a novel saturation mutagenesis approach; EMPIRIC (Exceedingly Meticulous and Parallel Investigation of Randomized Individual Codons) was exploited to investigate individual residues in a 20 amino acid region encompassing the CD4 binding loop (Fig 1A and 1F), for effects on replication and Env conformation. This 20 residue region includes conserved residues that contact CD4 (Fig 1B and 1C) and/or form part of the Phe-43 cavity (Fig 1D and 1E).

EMPIRIC involves the generation of libraries of mutations encoding all possible individual amino acid substitutions across important regions of genes [13–18]. Libraries are subject to selection or competition before analyzing by deep sequencing to quantify the frequency change of each mutation. Using EMPIRIC, substitutions in the CD4 binding loop and flanks were successfully identified that conferred enhanced or *wt* levels of replication in peripheral blood mononuclear cells (PBMCs). Several substitutions modulated the Env trimer with different mutations imparting distinct conformations that enhanced the exposure of the CD4bs but had varying effects on the TAD including the V3 loop and the glycan shield. One mutation enhanced the presentation of the quaternary trimer specific, V1V2 epitope (V2q) on the trimer apex that is recognized by PG9, PG16 and PGT145 monoclonal antibodies (mabs), consistent with a modified but closed trimer conformation. The effects of the different mutations were transferable to diverse clade B and C Envs. These observations confirm the capacity of EMPIRIC to identify single Env residues in the CD4 binding loop region that induce different conformational states in the TAD and trimer. This data is relevant for design of trimeric Env immunogens in vaccines that aim to protect against diverse HIV-1 clades.

## Results

### The primary LN40 Env and saturation libraries

Saturation mutant libraries were introduced into the primary LN40 Env [8, 19, 20]. LN40 env was PCR amplified and cloned from the lymph node of an AIDS patient with neurological complications. The LN40 R5 Env is not mac-tropic and is typical of Envs from immune tissue throughout disease [6, 21–23]. Most transmitted, founder R5 Envs are also not mac-tropic [24–27]. LN40 and other non-mac-tropic R5 Envs may form tightly closed trimers that protect against neutralizing antibodies (nabs) [28, 29]. Determinants of LN40 non-mac-tropism were previously mapped to residues on the N-terminal flank of the CD4 binding loop in addition to residues within V3. Presumably, these residues reduce access to CD4 (as well as nabs) and restrict replication to T-cells expressing high CD4 levels [8, 19, 20, 30]. It was predicted that mutations in the CD4 binding loop, its flanks and Phe-43 cavity would have strong potential to increase viral fitness by enhancing efficiency of Env/CD4 interactions [5, 8, 20, 31].

Two plasmid libraries were made containing all possible point mutations for Env amino acids 361–380 (361–370 and 371–380 in each library (See Movie at https://vimeo.com/165897330, password: Mama8) of LN40 env in full length, replication competent, pNL4.3 [21]. The vast majority of the mutants were present in the plasmid libraries and virions (P0) produced by transfection of 293T cells (S1A and S1B Fig). The frequency of most mutants in plasmid and P0 libraries was well above the background from all processing steps including RT, estimated by sequencing virus recovered from a plasmid with *wt* env (S1D Fig). The frequency of mutants in the P0 library correlated highly with that in plasmid library, indicating that P0 library recovery by transfection achieved a good sampling of mutants in the plasmid library (S1E Fig).

### Bulk competition of mutant libraries in PHA/IL-2 stimulated PBMCs

P0 viruses of each library were competed in bulk for amplification in PBMCs (S2A Fig). This process selects for viruses carrying wt and mutant Envs that are functional, but will overlook Envs carrying mutations that abrogate replication or that confer suboptimal replicative capacity. Nevertheless, this selection process enabled us to focus solely on mutations that did not have deleterious effects on replication. The abundance of each mutant was measured before and after amplification using Illumina deep sequencing and fitness estimated (see Materials and Methods). Stop codons were consistently depleted in both libraries (S2B Fig). All *wt*-synonyms in library-371-380 displayed *wt*-like fitness effects, although slightly more variation in fitness effects of *wt* -synonyms in library-361-370 was noted.

Following 8 days of infection, a strong correlation between enrichment and depletion of mutants in replicates was observed in library-371-380, but a weaker correlation between replicates in library-361-370 (S2D Fig). One explanation for the difference in reproducibility is that the virus stock containing library-361-370 had less infectivity compared to the stock containing library-371-380, and thus fewer virions mediated infection of PBMC. It was therefore more prone to insufficient sampling of P0 library, perhaps leading to stochastic enrichment or depletion of mutants.

Despite slightly higher variation in library-361-370, the results indicate that the majority of selection was reproducible and caused by introduced mutations. Data from the two replicates were pooled to obtain more precise measurements for analysis (S1 Table). The fitness effect of each amino acid was compared to that of repeatedly resampled *wt* -synonyms and an empirical p value of each amino acid computed for a fitness effect no different to that of *wt* -synonyms (Materials and Methods). After applying a 5% false discovery rate (FDR) as a multiple test correction, mutations were classified as statistically beneficial (significantly higher fitness than *wt*, synonyms), or statistically deleterious (significantly lower fitness than *wt*, synonyms), or statistically *wt*-like (neither beneficial nor deleterious) (S1 and S2 Tables). The majority of mutations in both libraries were strongly deleterious, indicating the 2 regions are extremely sensitive to missense mutations.

**Fig 2:**
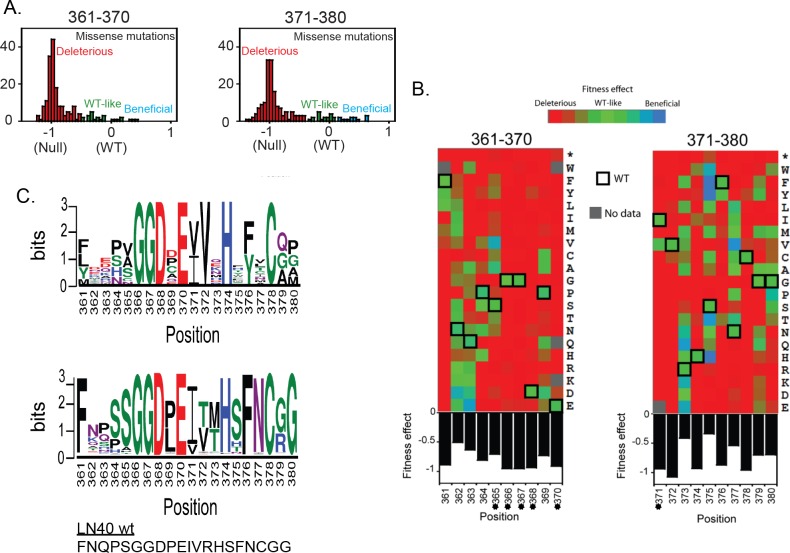
EMPIRIC analysis of the CD4 binding loop and flanks of LN40 Env and categorization of mutations as beneficial, wt or deleterious. (A) Most mutations were deleterious. However, subsets of mutations that imparted wt or higher levels of replication were identified. (B) The overall fitness effect of each substitution (average fitness effects at each position) is shown by designated colors (see key). Mutations that affect CD4 contact residues (marked with asterisks) were strongly deleterious. However, twelve mutations in regions downstream of the CD4 contact residues dramatically increased the efficiency of viral replication. These residues are proximal to the Phe-43 cavity. (C) The most common amino acids in positions 361–380 following PBMC passage of the two libraries (upper panel). The most common amino acids in positions 361–380 in all subtypes in the patient sequence database (http://www.hiv.lanl.gov/content/sequence/HIV/mainpage.html) (lower panel). The corresponding LN40 amino acid sequence is shown below the lower panel.

Both libraries had a limited number of mutants with *wt* -like fitness or above (S1 and S2 Tables; Fig 2A). Library-361-370 contained more fit mutations (19%) compared to library-371-380 (16%) even though it has less overall infectivity. This is also consistent with an increased variability of amino acids N-terminal to the CD4 contact residues on the HIV-1 (http://www.hiv.lanl.gov/content/sequence/HIV/mainpage.html). No other amino acids except *wt* residues were fully functional in CD4 contact residues (GGD_368__E_370_) (Fig 2B and 2C). In contrast, substitutions of proline at position P369 to cystidine, alanine, glutamine and aspartic acid conferred *wt* -like fitness. Positions 361–365 were more tolerant of mutations, especially positions 362 and 363, where the *wt* amino acids are asparagine and glutamine respectively. Charged amino acids (except for histidine) were well tolerated at these two positions (Fig 2B and 2C). At position 365, the *wt* is serine, whereas valine and alanine exhibited slightly increased fitness, although this was not significant.

In the 371–380 library, positions 373, 375 and 377 were tolerant of mutations. Even residues carrying side chains with different structures were at least *wt* -like in fitness (Fig 2B and 2C). For example, at position 373, glutamic acid was strongly beneficial over *wt* arginine. At position 375, where the *wt* amino acid is serine, all amino acids with aromatic rings as well as threonine and histidine were strongly beneficial. Glycine is the *wt* residue at position 380, and is relatively conserved in natural isolates (Fig 2C). Proline was slightly beneficial at 380. Positions 371, 372, 374, 376 and 378 were resistant to change, although substitutions to amino acids with very similar structures (e.g. Ile/Val and Phe/Tyr) were tolerated at some positions e.g. 371 and 376.

Overall, 13 beneficial mutations were identified, all in library-371-380 (S2 Table). Although a few mutations in library-361-370 exhibited small positive fitness effects, they were not statistically significant. This could be partly attributable to higher variation of fitness measurements in this library (due to lower overall infectivity) leading to reduced statistical power and/or to the optimal adaptation of this region to its function. All beneficial mutations in library-371-380 have a greater than 15% increase in fitness, which is very large compared to EMPIRIC studies in other systems [15].

### Fitness benefit and *wt* mutations identified by EMPIRIC result in changes in LN40 Env conformation and function

33 mutations that mostly conferred increased or wt levels of replication in PBMCs were investigated to elucidate effects on Env conformation (Table 1). The frequency of these mutant amino acids among the major HIV-1 subtypes are shown in S3 Table.

**Table 1:** The effect of LN40 mutations identified by EMPIRIC on Env structure and function. Neutralization assays were used to assess changes in Env structure and function

LN40 Env *wt* and mutants			sCD4	447-52D-*V3 crown*	b6-*CD4bs*	b12-*CD4bs*	17b-*CD4i*	PGT128	2G12-*glycans*
			IC50 fold change from LN40wt						
LN40 *wt*			1.00	1.00	1.00	1.00	1.00	1.00	1.00
361	F361I	d^1^	1.02	1.22	1.00	0.70	nt	1.14	1.68
	F361L	d	1.00	1.16	1.00	0.86	nt	1.14	1.49
	F361Y	w	1.40	1.33	1.00	0.70	nt	1.00	2.74
362	N362D	w	1.25	1.18	1.00	1.18	nt	1.00	0.56
	N362E	w	1.00	1.37	1.00	0.70	nt	1.00	1.49
	N362K	w	1.00	1.85	1.00	0.70	nt	1.14	4.73
	N362S	w	1.00	0.80	1.00	0.70	nt	1.33	2.74
	N362T	w	1.00	2.00	1.00	0.70	nt	1.00	2.60
	N362A	d	1.00	0.80	1.00	0.70	nt	1.14	2.74
363	Q363D	w	1.36	2.25	1.00	7.76	nt	1.00	0.47
	Q363E	w	1.44	2.21	1.00	13.96	nt	1.14	0.49
	Q363G	d	1.00	1.36	1.00	0.70	nt	1.14	0.83
	Q363H	d	1.84	2.76	1.00	0.70	nt	1.00	0.85
365^2^	S365A	w	1.56	1.84	1.00	0.70	nt	0.89	1.04
	S365V	w	1.12	1.40	1.00	0.70	1.00	1.00	1.00
369	P369A	w	1.00	0.80	1.00	0.70	nt	nt	0.58
	P369C	w	1.00	0.80	1.00	0.70	nt	nt	1.02
	P369D	w	1.00	1.54	1.00	0.70	nt	nt	0.37
	P369E	w	1.00	1.38	1.00	0.70	nt	nt	0.52
371^2^	R371V	w	1.00	0.80	1.00	1.01	nt	0.80	1.04
373	R373K	b	1.00	1.14	1.00	12.46	nt	0.89	0.63
	R373M	w	1.00	0.80	1.00	31.73	nt	0.89	0.69
	R373Q	b	1.13	1.59	1.00	23.27	nt	0.67	0.35
	R373E	b	2.09	16.67	1.00	17.45	1.00	0.67	0.20
	R373N	b	1.14	3.10	1.00	10.26	1.00	0.80	0.53
375	S375H	b	3.27	0.80	1.00	0.7	nt	0.57	0.33
	S375T	b	1.31	0.80	1.00	1.03	nt	0.89	0.62
	S375F	b	6.58	0.80	1.00	0.70	nt	0.62	0.24
	S375W	b	5.75	0.80	1.00	0.82	1.00	0.57	0.16
	S375Y	b	8.47	0.80	1.00	0.77	1.00	0.80	0.17
377	N377V	b	2.63	30.77	1.00	0.85	1.00	0.80	0.42
380	G380A	w	1.56	>200.0	1.49	1.30	1.00	0.80	0.46
	G380P	b	2.30	>200.0	3.27	1.38	1.00	0.40	0.20

1. b, beneficial; w, wt-like; d, deleterious substitutions.

2. CD4 contact residues.

nt; not tested.

green, >2,<4-fold; yellow, >4<100-fold; red, >100-fold differences.

Each mutation was introduced into the pSVIIIenv Env expression construct carrying LN40 env, before producing Env+ pseudoviruses (Materials and Methods). Changes in Env conformation were evaluated by testing sensitivity LN40 Env+ pseudovirions to inhibition by soluble CD4 (sCD4) and Env monoclonal antibodies (mabs) including CD4bs mabs, b6, b12, V3 loop mab, 447-52D, the glycan specific 2G12, V3 specific PGT128 and CD4i mab, 17b (Table 1).

#### Increases in sCD4 sensitivity

LN40 is relatively resistant to sCD4 inhibition due either to steric restrictions to binding and/or to resistance to CD4-induced conformational changes [30]. Sharp increases in sCD4 sensitivity for mutant Envs are consistent with changes in Env conformation that enhance CD4 interactions. Substitutions that increased sCD4 sensitivity included several at residue 375 e.g. S375W. Residue 375 is proximal to the Phe-43 cavity and S375W was reported to increase Env sampling of the CD4-bound form in gp120 monomers [5]. Substitutions at residues 373 (R373E), 377(N377V) and 380 (G380P) also conferred increases in sCD4 sensitivity. Other substitutions had less or no effect on sCD4 sensitivity (Fold change in IC50s are shown in Table 1 with IC50s in S4 Table).

#### Increases in sensitivity to CD4bs mabs

LN40 is resistant to the CD4bs mab b12. The b12 epitope is present on monomeric LN40 gp120 but occluded on the trimer [30]. Five substitutions at residue 373 (373E, 373M, 373N, 373Q and 373K) increased LN40 sensitivity to b12. This observation is not surprising since it was previously reported that the side chain of R373 (together with the glycan at N386) sterically restricted W100 of b12 from accessing a pocket on gp120 for binding [20].

All substitutions at 373 with a shorter side chain than arginine might be expected to expose the W100 pocket for b12 [20]. Substitutions Q363E and Q363D also conferred sensitivity to b12. These negatively charged residues may alter the structure of the b12 W100 pocket (e.g. by moving the glycan at N386) so that it is now open. Decreased 2G12 sensitivity supports a shift in the glycan shield including the glycan at N386, a 2G12 target [19]. The same 363 and 373 substitutions also impacted the TAD as detected by increases in 447-52D sensitivity indicating a more exposed V3 loop.

Mab b6 also targets the CD4bs. However, its epitope is occluded on primary Envs [7, 32]. Here, b6 failed to neutralize any mutant, except for G380A and G380P, which conferred weak sensitivity. This indicates that other substitutions tested did not sufficiently open the trimer to enable b6 binding.

#### Increased sensitivity to 447-52D and V3 loop exposure

Mab 447-52D recognizes a GPGR motif on the V3 crown which is occluded within the trimers of most primary HIV-1 strains, including LN40.

Substitutions that increased sensitivity to 447-52D, indicating a more exposed V3 loop, included Q363D, Q363E, Q363H, R373E and R373N. Substitutions at 377 (N377V) and 380 (G380A and G380P) also conferred enhanced sensitivity to 447-52D as well as to sCD4, consistent with a larger impact on the TAD. G380A and G380P mutants were exquisitely sensitive to 447-52D implying a more dramatic shift in the TAD and V3 loop exposure. In contrast, substitutions at residue 375 (e.g. S375W, S375Y and S375F) that conferred increased sCD4 sensitivity, remained resistant to 447-52D, indicating that the V3 loop was not exposed and that these changes induced a distinct trimer conformation.

#### Decreased sensitivity to 2G12 indicates a shift in the glycan shield

All the substitutions that increased sensitivity to sCD4 and/or CD4bs and V3 loop mabs also had modestly increased resistance to the glycan specific mab, 2G12, consistent with a change in the trimer conformation that shifts the orientation of the glycan shield.

#### Env mutants do not carry an exposed CD4i epitope

We focused on substitutions at 373, 375, 377 and 380, which imparted the most significant shifts in Env conformation and tested their sensitivity to the CD4i mab, 17b [2, 33]. We first confirmed that the epitope for mab 17b is present on LN40 by measuring binding to purified gp120 in the presence and absence of sCD4 (S3A Fig). It is unlikely that the library mutations themselves would affect the 17b epitope, which is focused on a distal bridging sheet site [2]. In neutralization assays, LN40 wt and each of the 373, 375, 377 and 380 substitutions were resistant to neutralization by 17b, thus indicating that changes in trimer conformation for these mutant Envs did not expose the CD4i epitope.

#### Several different residues substituted at P369 impart wt LN40 replication

CD4 contact residues, GGD_368__E_370_, in the CD4 binding loop were not readily substituted. However, mutations that substituted P369 with A, C, D and E residues imparted wt-like replication. P369 is relatively conserved in clade B Envs and its replacement with other amino acids might be expected to confer properties selected against in vivo. However, 369 mutants carrying A, C, D E, did not exhibit differences in sensitivity to sCD4, 447-52D, b6 or b12, while only D and E slightly decreased sensitivity to 2G12. These results indicated that this position accepts several different amino acids without detectable changes in Env conformation or replication fitness.

#### Summary of LN40 mutants

The use of multiple neutralizing mabs to investigate each substitution allowed an assessment of the changes distal to the residue in question and in overall Env conformation. The data presented show that different residues within or closely associated with the Phe-43 cavity have distinct effects on the conformation of the TAD and trimer.

### The effect of introducing an N160 glycan into LN40 Env

V2q mabs, PG9, PG16 and PGT145 bind the V2 glycan N160 and preferentially recognize the TAD at the apex of trimers rather than monomers. These mabs can be used as probes to assess whether this site has been disrupted consistent with trimer opening [30, 34]. Unfortunately, LN40 Env does not carry the N160 glycan, critical for binding V2q mabs, although a glycan at N156, also targeted by V2q mabs, is present. The N160 potential N-linked glycosylation signal was restored to LN40 Env by mutagenesis in the hope of reconstituting V2q mab binding. However, LN40 Y160N remained resistant to V2q mabs PG9, PG16 and PGT145 (S3B Fig) and presumably doesn’t bind these mabs.

**Fig 3:**
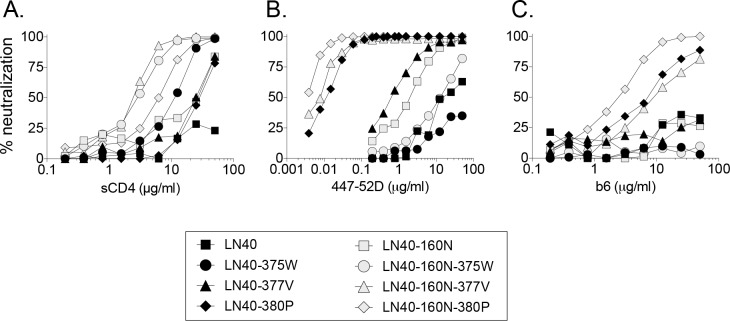
The effect of the Y160N substitution on LN40 wt and mutant Envs on sensitivity to sCD4 (A), V3 loop mab, 447-52D (B) and CD4bs mab, b6 (C).

Nevertheless, the introduction of N160 conferred increased sensitivity to sCD4 (Fig 3A, S5 and S6 Tables), consistent with a TAD conformation that enhances access to the CD4bs. Several substitutions identified by EMPIRIC conferred further enhancement in sensitivity to sCD4, the V3 mab 447-52D and CD4bs mab b6 when introduced together with Y160N (Fig 3A–3C, S5 and S6 Tables). In particular, LN40 160N mutants, 375W and, were more sensitive to sCD4 compared to Env mutants without Y160N. The presence of N160 also enhanced sensitivity of 377V to V3-specific 447-52D and more modestly to b6 (Fig 3B and 3C, S5 and S6 Tables). In contrast, the LN40 160N 375W mutant was only modestly more sensitive to sCD4 and remained relatively resistant to both 447-52D and b6.

These data indicate that the N160 glycan on LN40 conferred a trimer conformation where the CD4bs site is more exposed enabling enhanced interactions with CD4 and antibodies targeting V3 and the CD4bs.

### Mutations that change LN40 Env conformation conferred similar effects on another clade B Env and a clade C transmitter, founder Env

Several mutations were introduced into another clade B Env, LN8, and into a clade C Env, Z1792M. LN8 is an R5 envelope derived by PCR cloning from the lymph node of subject NA20, who died of AIDS with neurological disease [6, 21], while Z1792M is a transmitter, founder, clade C R5 Env from Zambia [35].

#### Substitutions at residue S375

**Fig 4:**
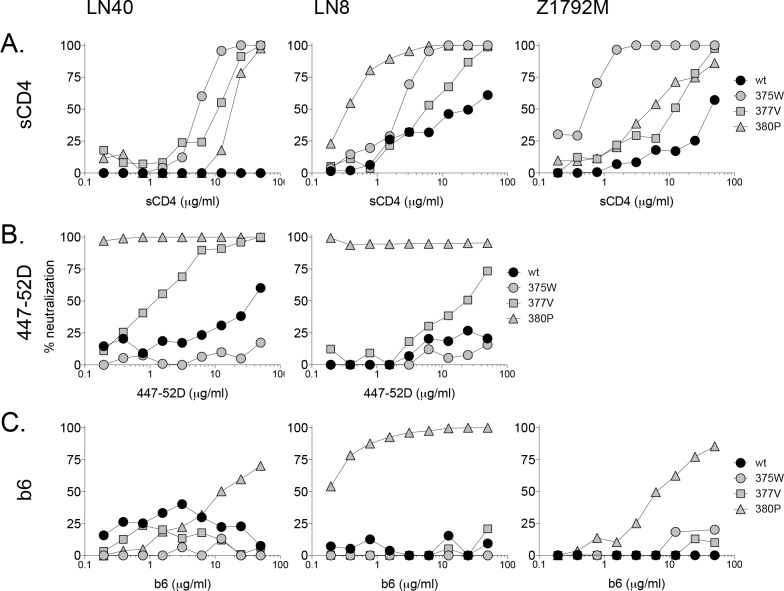
Substitutions at residues 375, 377 and 380 confer similar effects on clade B LN40, LN8 and clade C Z1792M Envs. Neutralization profiles for sCD4 (A), 447-52D (B) and b6 (C) are shown. Each substitution had similar effects on the three different Envs. consistently increasing sensitivity to sCD4. However, only 380P conferred sensitivity to mab b6, while 377V and 380P exposed the V3 loop crown (447-52D epitope), while 375W didn’t. Of note, Z1792M does not carry the 447-52D epitope.

Substitutions S375H and S375W both enhanced sCD4 sensitivity of LN8 and clade C, Z1792M, as they did for LN40 (Fig 4A; S7–S10 Tables). These S375 substitutions had little effect on LN8 sensitivity to b12, b6 and 447-52D, yet conferred more resistance to 2G12. This is consistent with a shift in the orientation of one or more glycans resulting in a more exposed CD4bs and increased sCD4 sensitivity. The changes implicated in LN8 Env conformation closely follow those described above for LN40 Env. Z1792M does not carry the b12, 447-52D or 2G12 epitopes. However, S375 substitutions had no effect on b6 resistance.

#### The N377V substitution

N377V conferred increased sensitivity to sCD4 for LN8 and Z1792M and modestly increased sensitivity of LN8 to V3 mab, 447-52D, following observations made for the equivalent LN40 mutant (Fig 4A and 4B).

#### Substitutions at G380

G380P enhanced sCD4 and b6 sensitivity for LN8 and conferred exquisite sensitivity to the V3 mab, 447-52D. G380P also rendered LN8 highly sensitive to mab b12, in contrast to LN40 where there was no effect. In addition, G380P enhanced sensitivity to sCD4 and b6 for Z1792M (Fig 4, S7–S10 Tables). These results indicate that G380P conferred more dramatic changes to the trimer conformation of LN40, LN8 and Z1792M Envs resulting in improved CD4 interactions and enhanced exposure of the V3 loop (at least for LN40 and LN8) and b6 epitope, consistent with a more open trimer conformation. Together, these findings indicate that substitutions at 375, 377 and 380 enhance Env/CD4 interactions across two diverse clades.

#### L369P substitution in clade C Z1792M Env

P369 is conserved on the clade B database, while L369 is dominant for clade C. A L369P substitution was made in clade C, Z1792M Env. The presence of P369, rendered Z1792M more sensitive to sCD4 (S9 and S10 Tables), perhaps indicating an Env trimer more open to nabs and possibly explaining the selective advantage of L over P at 369 for clade C Envs in patients.

### The effects of Env substitutions on the V2q epitope of LN8 and Z1792M

**Fig 5:**
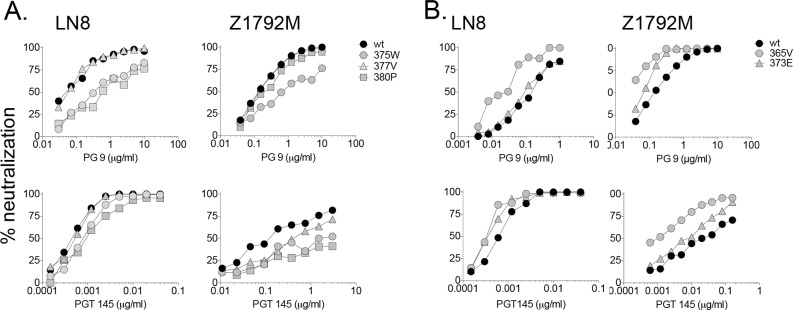
Env substitutions in the CD4 binding loop modulate the trimer specific, V2q epitope. (A) Env substitutions proximal to the Phe-43 cavity, reduced sensitivity to V2q, mabs, PG9 and PGT145, consistent with a more open TAD and trimer. (B) Substitution S365V on the variable, N-terminal flank of the CD4 binding loop enhanced sensitivity to V2q mab PGT145, consistent with a modified (but not more open TAD conformation).

Unlike LN40, LN8 and Z1792M Envs carry the glycan at N160 in V2 and are sensitive to the trimer specific V2q mabs PG9 and PGT145 [36]. Measuring sensitivity to V2q mabs could help monitor whether trimers are closed for each mutant. Surprisingly, all LN8 and Z1792M substitutions remained sensitive to PG9 and PGT145, although Envs carrying S375W, N377V and G380P substitutions in LN8 and Z1792M were modestly less sensitive to either PG9, PGT145 or both (Fig 5A and S7–S10 Tables). To verify that the V2q, trimer apex epitope could be disrupted, we measured PG9 binding to LN8 trimeric Envs expressed on 293T cells in the presence or absence of sCD4. We chose the LN8 S375W mutant Env for this control experiment since it is highly sensitive to sCD4 inhibition. S4 Fig shows that prior treatment of the LN8 375W mutant Env with sCD4 led to a dramatic reduction in PGT145 staining consistent with the trimer opening and the V2q, trimer apex abrogated.

The modest impact of the S375W, 377V and 380P substitutions on sensitivity to V2q mabs is curious. How can the LN8 G380P Env trimer be open sufficiently to bind b6 and 447-52D, yet remain substantially sensitive to V2q mabs, PG9 and PGT145 that recognize closed trimers? One possibility is that PG9 or PGT145 bind open trimers by retaining an interaction with monomeric gp120. However, using ELISAs, it was confirmed that these mabs do not bind Z1792M gp120 monomers (S3C Fig). It is therefore likely that the native trimers of mutants 380P and 377V take up a conformation where the PG9, PGT145 epitope in the TAD is largely maintained while still exposing the V3 loop crown and CD4bs including the b6 epitope (S5A Fig). However, there is an intriguing alternative where these mutant trimers transition to and from closed and open forms [37], so that b6 captures the open state, while V2q mabs capture the closed state (S5B Fig).

### An S365V substitution in a CD4 contact residue enhanced the V2q epitope

While substitutions downstream of the GGD-E (366–370) CD4 contact residues, resulted in changes consistent with TAD and trimer opening, mutations in upstream residues had little effect on sensitivity to sCD4 and other mabs (Table 1 and S4 and S7–S10 Tables). Exceptions include N362D, Q363D and Q363E, which enhanced b12 sensitivity for either LN8 or LN40.

An S365V substitution modestly increased sensitivity to both PG9 and PGT145 for Z1792M. Effects on LN8 were more marginal, although S365V was still the most sensitive LN8 mutant for PG9 and PGT145 (Fig 5B, S7–S10 Tables). The 373E substitution also enhanced (rather than reduced) the V2q epitope, although less than for S365V.

### Investigation of mutant Env trimer conformation in a direct binding assay

**Fig 6:**
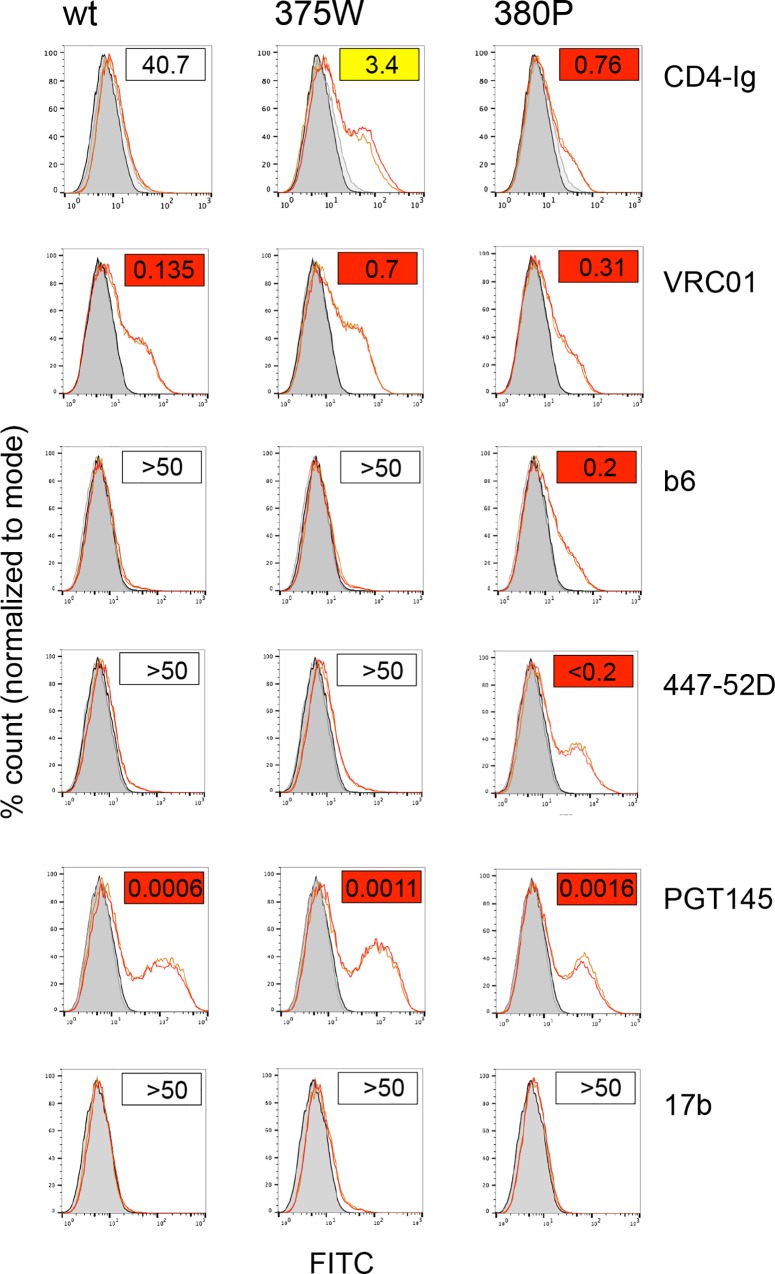
Direct binding measurements of CD4-Ig and mabs follows neutralization sensitivity of Env+ pseudoviruses. LN8 wt, 375W and 380P Envs were expressed on 293T cells before measuring binding of CD4-Ig and mabs using flow cytometry. Boxed values in the right hand, top corner of each flow profile represents the neutralization titer for each reagent and shows that binding closely followed neutralization sensitivity.

Experiments described above exploited neutralization assays with Env-specific mabs to investigate the exposure of different epitopes. However, while mab binding is a critical step for neutralization, other mechanisms may also be involved [38]. We next used a direct mab/trimeric Env binding assay to confirm critical data for LN8 mutants 375W and 380P. LN8 wt and mutant Envs were expressed on 293T cells before evaluating mab binding by Flow cytometry. Data presented shows that binding of CD4-IgG and mabs VRC01, b6, 447-52D, PGT145 and 17b accurately followed neutralization measurements (Fig 6).

### Sensitivity to potent CD4bs mabs and macrophage-tropism

The ability of HIV-1 R5 strains to infect macrophages has been shown to reflect an enhanced Env:CD4 interaction [6, 21, 39, 40]. Several of the Env mutants described above (e.g. 375W, 377V and 380P) were more sensitive to sCD4 inhibition, consistent with a more efficient Env:CD4 interaction. Neutralization data using CD4bs mabs, b12 and b6 also supported a more exposed CD4bs for several mutants. We next tested the sensitivity of LN40, LN8 and Z1792M Env mutants (375W, 377V and 380P) to the highly potent CD4bs mabs, VRC01 and 3BNC117 (S11 Table). These CD4bs mabs bind gp120 monomers as well as closed and open trimers [41, 42]. They both recognize a very broad range of diverse HIV-1 Envs. Here, VRC01 and 3BNC117 mabs neutralized LN8 and LN40 wt Envs as well as 375W, 377V and 380P Env mutants as expected confirming that the 375W, 377V and 380P Env mutants have not compromised the VRC01-related epitope while exposing the CD4bs. Nonetheless, both LN40 and LN8 mutants showed slightly reduced sensitivity to both VRC01 and 3BNC117 mabs, which preferentially favor binding to tightly packed, closed trimers [41]. These results are consistent with data from other CD4bs mabs (b12 and b6) and support more open trimers for the mutant Envs. Z1792M did not bind either VRC01 or 3BNC117. We also tested mab 8ANC195 which binds to a conserved epitope at the junction of gp120 and gp41 and recognizes both open and closed trimers [43]. 8ANC195 only recognized Z1792M, neutralizing wt as well as 375W, 377V and 380P similarly (S11 Table).

**Fig 7:**
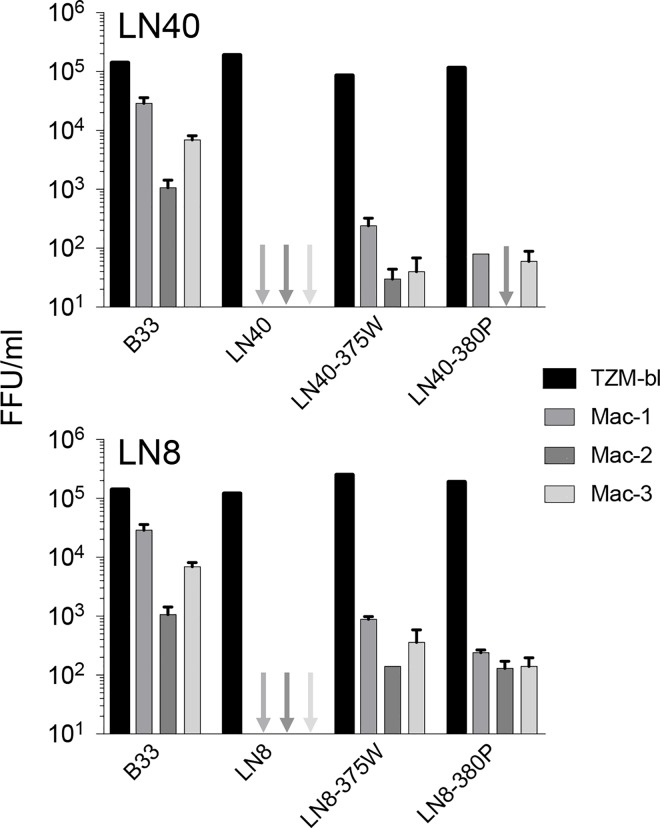
Macrophage infection mediated by LN40 and LN8 Env mutants 375W and 380P. Macrophage infectivity was measured as described in Materials and Methods using monocyte derived macrophages from three independent donors. 375W and 380P mutants (but not wt) Envs conferred infection of macrophages by Env+ pseudoviruses. However, infectivity was substantially lower than that of a pseudovirus carrying B33 Env, an R5 mac-tropic Env.

Finally, we tested whether LN40 and LN8 Env mutants (375W and 380P) could trigger infection of primary macrophages. Pseudoviruses carrying Env mutants 375W and 380P (which exhibit enhanced sensitivity to sCD4), were tested for infection of monocyte-derived macrophages from three different donors. Each of the mutant Envs mediated macrophage infection, while the wt LN40 and LN8 Envs did not (Fig 7). Nevertheless, the levels of macrophage infection mediated by the mutant Envs were substantially lower than that recorded for the mac-tropic R5 Env, B33 (from the same subject as LN40 [6, 21]).

These observations are consistent with a more exposed CD4bs and more efficient Env:CD4 interactions. However, they indicate that further substitutions would be required to confer full macrophage infectivity.

## Discussion

An HIV-1 vaccine that induces potent and broad nabs will require detailed knowledge of the residues that control the configuration of the Env trimer. This information will help design Env immunogens that optimally present the best epitopes for eliciting the most rigorous vaccine response.

A novel saturation mutagenesis approach, EMPIRIC, was applied to investigate the role of residues in a 20 amino acid stretch of gp120 in regulating the conformation of the TAD and trimer. This region encompasses the CD4 binding loop and flanking regions. Several residues were identified that modulated the TAD and trimer (summarized in Table 2, S6 Fig and Movie, https://vimeo.com/165897330, password: Mama8). However, different residues enhanced Env: CD4 interactions by imparting distinct TAD conformations with differential exposure of the V3 loop and other epitopes usually occluded within the trimer. The majority of mutations that modified LN40 TAD and trimer had similar effects on LN8 and clade C, Z1792M, indicating that residues identified, control Env conformation across clades. Several residues in this region have been examined previously. However, our data represents a large new resource for this region of Env and greatly extends previous studies. For example, S375F, S375Y and S375W were studied in the context of monomeric gp120 [5], while 375 substitutions into functional Env trimers mainly focused on S375W [5, 31]. More recently, Li et al. showed that 375 variants carrying bulky hydrophobic side chains in SHIV enhanced interactions with macaque CD4 as well as their replication and pathogenesis in macaques [44].

**Table 2:** Summary of Env mutations that affect trimer conformations.

Env region	Substitution	Specificity	Affected Env region			
			CD4bs	V3 loop^1^	TAD V2q epitope^2^	2G12 glycans
V2 loop	160N	Only LN40 studied	more exposed to sCD4 but not CD4bs mabs	more exposed	not tested	not tested
CD4 binding loop, N-terminal flank	362D	LN8 specific	mab b12 epitope more exposed	hidden	no effect	shifted
	363D	LN40 specific	mab b12 epitope more exposed	more exposed	not tested	shifted
	365V	Z1792M, LN8 more marginal	No effect	still hidden	enhanced presentation	no change
Phe-43 cavity	375W	cross clade^3^	more exposed	still hidden	reduced	shifted
CD4 binding loop, C-terminal flank	377V	cross clade^3^	more exposed	exposed	reduced	shifted
	380P	cross clade^3^	more exposed	highly exposed	reduced	shifted

1 Not tested on Z1792M.

2 Not tested on LN40

3 V3 exposure not tested on clade C, Z1792M

Mutations likely to affect trimer conformation were identified in competition assays by replication in PBMCs. This approach selects for mutations that support viral replication in the absence of nabs and enabled the identification of residues infrequent on the HIV sequence database. The effect of selected mutations on Env conformation was then investigated using a panel of Env mabs which recognized a range of Env epitopes. We focused initially on mabs that recognized epitopes that are usually occluded on native trimers including 447-52D (V3 loop), b6 (CD4bs) and 17b (CD4i) as well as assessing shifts in sensitivity to sCD4 inhibition. This approach thus measures the exposure of epitopes and should not be greatly impacted by library mutations destroying specific mab epitopes. For example, substitutions 375W, 377V and 380P were shown to increase CD4 binding and result in more open trimers where usually occluded epitopes are exposed. Two types of substitution were observed. (1) Substitutions that modified the local structure around the CD4bs e.g. N362D in LN8, where the b12 epitope was exposed without affecting other sites. (2) Substitutions that modified distal sites including V3 and the TAD e.g. S365V, S375W, N377V and G380P. One concern with the competition assay was whether spontaneous mutations contributed to enhanced replication. This was not investigated. However, many mutations mediated significant effects on the trimer when investigated individually using pseudoviruses carrying mutant Envs. These observations confirm that EMPIRIC identifies physiologically relevant amino acid substitutions and is a formidable mutagenesis tool for analyses of Env conformation, fitness or other properties, relevant for vaccines.

Two libraries were prepared covering residues 361–370, and 371–380 (Fig 1). Library-361-370 contained the variable N-terminal flank [30] and CD4 contact residues GGD_368_-E_370_. Few mutants from this library were identified that increased replicative capacity over *wt* virus or conferred a more open Env conformation. Nevertheless, many variant residues on the N-terminal flank conferred wt-like replication, contrasting with mutations in conserved CD4 contact residues where *wt* residues predominated during PBMC replication (Fig 2B and 2C). These observations indicate that these latter amino acids have been highly selected during evolution as would be predicted for sites contacting a major receptor. The lack of beneficial mutants in the variable 361–365 region was curious, since a motif on this variable flank region was previously identified that influenced the non-mac-tropic phenotype of LN40 Env [8]. However, this motif required the presence of further determinants in V3 to mediate maximal effects on LN40 Env properties [8].

A small number of substitutions on the N-terminal flank did affect Env properties. LN40 Q363D and Q363E mutants were more sensitive to the CD4bs mab, b12, with more modest effects on sCD4, 447-52D and 2G12 sensitivity detected. The 365V substitution conferred a small (not significant) increase in replication for LN40 Env+ virus in PBMCs. When introduced into other Envs, an increase in sensitivity to the trimer apex mabs, PG9 and PGT145, was detected for Z1789M (and more marginally for LN8) consistent with effects on the conformation of the TAD but without detectable opening of the trimer.

Residue 369 is a relatively conserved residue situated between CD4 contact residues (GGDP_369_E) at the tip of the CD4 binding loop. There is some clade specificity to this residue i.e. P in clades B and D, L in clades A and C. In clade B LN40, several substitutions were tolerated without detectable effects on Env properties. In contrast, an L369P substitution in clade C Z1792M resulted in an enhanced sensitivity to sCD4 consistent with increased exposure of the CD4bs. These data suggest that variation at residue 369 is selected against in vivo as it may result in increased exposure of the CD4bs to nabs, at least for clade C. However, no insights were obtained into the selective pressures that prevent 369 variation in clade B.

Several residues in library-371-380 could be replaced by residues that enhanced replication in PBMCs. These residues had strong effects on the conformation of the trimer and TAD as indicated by significant increases in sensitivity to sCD4, CD4bs mabs b6 and b12, and to V3 crown mab 447-52D depending on substitution. The positions in 371–380 are each closely associated with the Phe-43 cavity and likely play a role in controlling Env conformational changes in response to CD4 binding [3, 4].

Residues that altered the TAD and trimer conformations had different effects on the exposure of mab epitopes. Several residues in the 371–380 region conferred enhanced sCD4 sensitivity, yet affected exposure of the 447-52D V3 and b12 epitopes differently. Thus, G380P increased sensitivity to sCD4 in LN40, LN8 and Z1792M, and also imparted greatly enhanced sensitivity to 447-52D for both LN40 and LN8. However, while G380P conferred sensitivity to b12 for LN8, there was little effect for LN40. S375W, S375Y and S375F mediated increases in sCD4 sensitivity for LN40, but had no effect on 447-52D resistance. Thus different residues associated with the Phe-43 cavity, regulate distinct conformations of the TAD at the trimer apex and highlight a potential role for this region in regulating immune protection. It is also worth noting that the 375W, 377V and 380P substitutions did not affect the integrity of the VRC01/3BNC117 CD4bs epitope, a highly desirable target for the induction on potent neutralizing antibodies. Together, these observations thus have relevance for vaccine development, where Env trimers fine-tuned for enhanced exposure of the CD4bs but without exposing irrelevant immunogenic sites (e.g. the V3 loop) that are occluded on primary Envs are desired immunogens.

For several mutants that had modified TAD and/or trimers with enhanced exposure of the CD4bs, it was noted that sensitivity to 2G12 was reduced. Mab 2G12 recognizes several glycans on gp120 and its ability to bind and neutralize virions depends on their orientation. Reduced sensitivity to 2G12 suggests that modified, more open Envs have shifted the glycans forming this mab’s epitope as previously reported [19].

The glycan N160 in V2 is a target for V2q antibodies and its lack explains why LN40 Env is insensitive to PG9 and PGT145 mabs. This was unfortunate since V2q mabs can be used to investigate changes in TAD configuration. Introduction of the potential glycan site at N160 in LN40 Env failed to restore sensitivity to V2q mabs. However, the presence of the glycan site at N160 increased sensitivity to sCD4 and V3 mab, 447-52D. These observations indicate that in the context of LN40, N160 modified the trimer conformation, exposing the V3 loop and increasing access for CD4. N160 also enhanced the effects of other substitutions on Env conformation. For example, 373E, 377V and 380P were all more sensitive to V3 loop mab, 447-52D in the presence of N160. N160 on LN40 may loosen the TAD and facilitate some substitutions to confer more extensive shifts in conformation. It is worth noting that the presence of N160 had only marginal effects on the S375W substitution, with low level 447-52D neutralization detected and sCD4 inhibition slightly enhanced. These observations add further support to the conclusion that S375 substitutions induce a trimer conformation distinct from those imposed by other substitutions e.g at 377, 380. The increased exposure of the CD4bs and V3 loop following the introduction of an N160 glycan to LN40 is curious. It suggests that LN40 Env evolved a distinct native conformation that is unfavorable for an N160 glycan. It would be interesting to assess whether other primary Env trimers that lack an N160 glycan, also expose the usually protected CD4bs, V3 epitopes if one is introduced.

It was surprising that several mutant LN8 and Z1792M Env trimers were sufficiently open to bind mabs b6 and (for LN8 mutants) 447-52D, yet remained sensitive to V2q mabs (that predominantly recognize closed trimers), albeit with modestly increased IC50s. It is therefore likely that some substitutions can expose the b6 epitope in the CD4bs region as well as the 447-52D epitope without significantly opening the TAD (S5 Fig). However, an intriguing alternative explanation is that Envs transition from closed to open states and back, so that mabs b6 and 447-52D capture the open form, while the V2q mabs capture the closed form.

LN40, LN8 and Z1792M wt Envs are non-mac-tropic and require high levels of cell surface CD4 to trigger fusion and entry. The enhanced Env: CD4 interactions detected for several Env mutants would be expected to increased their capacity to mediated macrophage infection. This possibility was evaluated for LN40 and LN8 375W and 380P substitutions with a modest enhancement of macrophage infection observed. However, this level of macrophage infection was substantially lower in comparison to that conferred by a highly mac-tropic brain R5 Env (B33). This result indicates that further mutations would be required for a full mac-tropic phenotype. Whether, such additional mutations would further enhance Env: CD4 interactions or affect macrophage infection via a distinct mechanism is unclear.

In summary, mutations in the CD4 binding loop and flanks that affect trimer conformation were identified in replication competition assays. Different substitutions identified were associated with distinct conformations that impacted on the exposure of the V3 loop and TAD, the CD4bs and the efficiency of CD4 interactions in Envs from different clades. The information presented contributes to the establishment of universal, cross clade rules for regulating trimer conformation and will be invaluable in the design of next generation Env immunogens.

## Materials and Methods

### Construction of plasmid-encoded libraries

Env saturation mutant libraries were generated using a previously described approach [13, 15]. Briefly, the env gene was cloned into pRNDM to generate a plasmid without BsaI restriction sites. Inverted BsaI sites were then introduced to allow for a cassette ligation strategy with each single codon randomized as NNN to efficiently generate libraries of all possible codon substitutions; a separate cassette was used to mutate each codon to all 63 non-parental ones. Libraries of single codon mutants at 10 consecutive codons were combined and the resulting pool transferred from pRNDM to replication competent pNL4.3 with LN40 env, using sequence and ligation independent cloning (SLIC) [45]. A SLIC destination vector was generated that encoded the HIV genome with the majority of the env gene removed and a unique BmtI restriction site at this location. The destination vector was digested with BmtI and resected with T4 DNA polymerase as described previously [45] to leave approximately 30 bases of single stranded DNA at each end. Linear fragments of the env libraries from pRNDM with single stranded regions matching the prepared destination vector were generated by PCR (using Pfusion high fidelity polymerase and eight cycles of amplification to minimize amplification errors) and treatment with T4 DNA polymerase. The prepared library and destination DNA were mixed at equal molar amounts, annealed for 30 minutes at 37°C, and transformed into bacteria to generate the plasmid libraries encoding full-length viral genomes.

### Viral library recovery and competition experiments

2.5 μg of DNA encoding full length NL4.3 carrying LN40 or 361–370 and 371–380 mutant library envelopes were transfected into 293T cells using calcium phosphate. Supernatants carrying full length NL4.3-LN40env or libraries (P0) were harvested 48 h after transfection, clarified (1,000*g* for 10 min), aliquoted, and stored at -152°C. HeLa TZM-bl cells were used to titrate the P0 stock libraries using the LTR-controlled β-galactosidase reporter gene to identify infected cells as described previously [46].

20x10^6^ PHA treated peripheral blood mononuclear cells (PBMCs) were recovered from a frozen stock and infected with 2 ml wild type (wt) LN40 virus, or with each library virus stock (P0) in duplicate. After 3 hours, infected PBMCs were centrifuged at 1200 rpm for 10 min. Supernatant was harvested and frozen as day 0 (D0). Cells were washed with 5ml of RPMI/10% fetal calf serum twice before adding 10 ml RPMI/10% fetal calf serum with IL-2 (Roche Inc.). Medium was changed after 4 days and supernatants collected on day 8. 200 μl samples were treated with recombinant DNase I for 2h at room temperature to eliminate any carry over of plasmid DNA before extracting RNA using the High Pure viral RNA kit (Roche Inc.).

### Sequence analyses and estimation of fitness

HIV genomic RNA was extracted from supernatants containing virions using High Pure Viral RNA kit (Roche Inc.). Viral RNA was reverse transcribed into cDNA using primers binding downstream of randomized libraries and SuperScript III (Life Technologies Inc.). Subsequent processing steps were as described previously for analyzing mutant frequency [13]. Briefly, samples were barcoded to distinguish replicates as well as plasmid, P0, and P1 samples and submitted for Illumina 36bp single read sequencing on a Genome Analyzer II. Reads with a phred score of 20 or above (>99% confidence across all 36 bases) were analyzed (S12 Table). The relative abundance (*A*) of each point mutant of plasmid, P0 and P1 library was estimated from read abundances (*R*) as indicated below in Eq (1).

$$A=\log_{2}\left(\frac{R_{mut}}{R_{WT}}\right)$$ (Eq. 1)

The frequency change (*F*) of a mutation from P0 to P1 (Eq 2) was used as an estimate of the enrichment or depletion during viral expansion. Two replicates of P1 were determined separately.

$$F=A_{P1}-A_{P0}$$ (Eq. 2)

Selection coefficients (*s*) representing the experimental effects of each mutation were calculated by normalizing the median of stop codon to -1 (representing null fitness) and wild-type synonyms to 0 (representing no fitness effect), as indicated in Eq (3).

$$s=\frac{F_{mut}-F_{\bar{WTsyn}}}{F_{\bar{WTsyn}}-F_{\bar{stop}}}$$ (Eq. 3)

The above analyses yielded estimates of fitness effects for each codon with frequency > 0.015% in the P0 library (S12 and S13 Tables). Mutations below this frequency in P0 were likely subject to highly stochastic sampling in the pool of viruses used to start P1 passages. These mutations (4% of the data) also had very low frequency in the plasmid library. The high correlation between frequency of mutants in P0 and the plasmid library suggested that viral recovery by transfection provided sufficient sampling of mutants in the plasmid library (S1E Fig), so mutants with low frequency in P0 library was due to their inherent low frequency in the plasmid library and not due to a bottleneck effect or selection in viral recovery.

As estimates of selection coefficient (s) had some noise, in particular in the 361–370 library (S2C and S2D Fig), the median of s of synonymous codons encoding the same amino acid was used to represent s of that amino acid, to minimize impact from outliers. RMSD was determined between the two replicates to estimate variation in s of amino acids. The two replicates in P1 were then pooled to estimate the selection coefficient of amino acids to further improve reproducibility. Specifically, the median was computed for s of all synonymous codons encoding the same amino acid in both replicates as s for that amino acid (S1 Table).

To determine whether a mutant is statistically beneficial, or *wt* -like, or deleterious, the s of each amino acid was compared to the median of s of resampled *wt* -synonyms, and defined mutants with s significantly greater than that of *wt* -synonyms as beneficial; mutants with s significantly less than that of *wt*-synonyms as deleterious and the rest as *wt* -like. All *wt*-synonyms of both replicates were pooled for each library (38 for Library-361-370 library and 50 for Library-371-380 library). For each amino acid, *wt*-synonyms were resampled as twice the number of synonymous codons encoding that amino acid and compared the median of s of resampled *wt*-synonyms with s of that amino acid. This process was repeated 10,000 times for each amino acid and computed the proportion (f) when s of amino acid is greater than median s of resampled *wt* -synonyms. 1-f (if f>0.5) or f (if f< = 0.5) is the empirical p value of this amino acid having a fitness effect greater than or less than *wt* -synonyms. Before multiple test corrections, mutants with p value <0.025 have a significantly different s with *wt* -synonyms (S1 Table). A two-sided 5% False Discovery Rate (FDR) was then applied as multiple test correction. After that, amino acid mutants with a sufficiently small p value were classified as statistically beneficial or deleterious and the rest as statistically *wt* -like. False negative rates were not estimated, so that a small number of mutants that were classified as *wt*-like might be beneficial or deleterious. Amino acids with more synonymous codons were treated as having more replicates so that they would have stronger statistical power in classification.

### Cloning of individual mutants

A panel of individual gp120 mutations were cloned into the pSVIIIenv vector that carried clade B LN40, LN8 or clade C Z1792M env genes and analyzed in isolation. A cassette with a single mutant was ligated into BsaI digested pRNDM, as described above, and then subcloned into pSVIIIenv. For LN40 env, one KpnI site in pSVIIIenv vector outside env coding region, was eliminated with quick change mutagenesis. pRNDM with mutants and pSVIIIenv were both digested by KpnI and SpeI, and the mutant fragments from pRNDM ligated into digested pSVIIIenv. For LN8 env, two sets of primers were utilized to generate two fragments of env by PCR. The 3’ region of one fragment shared a 27-nucleotide homologous region with the 5’ region of the other fragment, and the 3 nucleotides in the middle of the homologous region were mutated to desired mutations by PCR. Primers are described in S14 Table. pSVIIIenv was digested by KpnI and SpeI, followed by T4 polymerase trimming to generate matched ends ready for homologous recombination. The digested vector and the two PCR generated fragments were then assembled back into the full length pSVIIIenv vector through Gibson assembly to generate a set of mutants (New England Biolabs, Ipswich, MA).

### HIV Env clones, sCD4 and monoclonal antibodies

EMPIRIC libraries were cloned into pNL4.3 carrying the LN40 env gene. A version of LN40 env was used that was chimeric with LN40 gp120 and gp41 sequences derived from the B33, a brain env derived from the same subject as LN40. This chimeric Env is non-mac-tropic and carries determinants and properties of non-mac-tropism in gp120 as reported previously [6–8, 21].

Soluble CD4 was from Progenics Inc. Monoclonal antibodies (mabs) PG9, PG16 (V2q), b12 (CD4bs), 447-52D (V3 loop) and 2G12 (glycans) were from Polymun Scientific Inc. (Austria). PGT145 (V2q) and VRC01 (CD4bs) were prepared in house. Mab b6 (CD4bs) was provided by Dennis Burton (Scripps Research Institute), mab 17b (CD4i) by George Lewis (Institute of Human Virology) and mab PGT128 (V3, glycans) was provided by IAVI. 3BNC117 [42] was provided by the NIH AIDS Reagent Program.

### Preparation of Env+ pseudoviruses

Env+ pseudotypes were produced and titrated as described previously [6, 21, 24, 47]. Briefly, Env+ pSVIIIenv constructs carrying different single mutations were cotransfected into 293T cells with *env*-minus pNL43. Env+ pseudovirions were harvested after 48 hours, clarified by low speed centrifugation and frozen as aliquots at -152°C. Env+ pseudovirions were titrated on HeLa TZM-bl cells cells, which carry β-galactosidase and luciferase reporter genes controlled by HIV LTR promoters [46]. Infected cells were visualized 48 hours after infection as focus forming units (FFU) following staining for β-galactosidase activity. Since Env+ pseudovirions are only capable of a single round of replication, individual cells or small groups of divided cells were counted as foci.

### Antibody neutralization assays and IC50 determination

Neutralization and inhibition assays were performed as described previously using 200 FFU of Env+ pseudovirus and evaluating residual infectivity on HeLa TZM-bl cells via a luminescence readout [7, 30].

### Detection of the epitope of mab 17b on monomeric gp120 by ELISA

*Production and measurement of LN40 gp120 concentration*: Monomeric LN40 wt gp120 was prepared by transfection of pJW4303-LN40 gp120 into 293T cells using 293Fectin (Thermo Fisher Scientific Inc.). Cell supernatant was harvested 72 hours after transfection and purified by lectin column chromatography [48]. The concentration of gp120 was estimated by titration using a capture enzyme-linked immunosorbent assay (ELISA) by comparing the half maximal binding dilution of LN40 gp120 to a standard concentration of IIIB gp120 [49].

*Mab 17b binding to monomeric gp120*: 17b binding to LN40 gp120 was evaluated using a modified capture ELISA. Briefly, saturating amounts of LN40 gp120 were captured onto ELISA plates treated with sheep anti-gp120 antibody (Aalto Bio Reagents Inc.) [30, 49], before testing 17b (5 μg/ml) binding in the presence of increasing concentrations of sCD4.

### Expression of Env trimers on 293T cells and mab binding

HIV-1 Envs were expressed on 293T cells following transfection of Env expression vectors using Fugene6 following the manufacturer’s protocol. Briefly, Envs were expressed from pSVIIIenv by co-transfecting with pSV2-Tat72 and pGEMfurin [50]. Env expression was evaluated 2 days post transfection using a panel of Env mabs to distinguish the predominant expression of cleaved trimeric Env over uncleaved or defective Env forms [50]. In particular, mabs PG9 and PGT145 (V2q, trimer specific), 447-52D (V3 loop), b6 (CD4bs), 17b (CD4i) were used to detect closed trimers where the V3 loop and CD4i epitopes are occluded [50]. We also tested several other mabs as described above, as well as CD4-Ig. Binding of these human mabs and CD4-Ig was detected with an anti-human IgG-FITC conjugate and examined by flow cytometry in the University of Massachusetts Medical School Flow Cytometry Core.

### Preparation of primary macrophages and their infection

5x10^7^ peripheral blood mononuclear cells (PBMCs) from a buffy coat (New York Biologics) were plated into 14-cm bacterial culture dishes for 3 h before extensively washing away non-adherent cells, culturing overnight, and repeating the washes. The adhered monocytes were then cultured for 5 to 7 days in 10% AB+ human plasma in DMEM before treatment with EDTA and transfer to 48-well tissue culture dishes the day prior to infection seeding at 1.25 x 10^5^ cell/well [6, 21, 24].

Macrophage infectivity of Env+ pseudoviruses carrying LN8 and LN40 wt and mutant (375W and 380P) Envs was assessed on duplicate wells of 3 batches of macrophages prepared from independent donors. Macrophages (seeded in 48 well plates) were pretreated with 100 μL DEAE dextran (10 μg/mL) in DMEM medium containing 10% human plasma for 30 min at 37°C before Env+ pseudoviruses were added at 100 μL/well. Infected plates were spinoculated for 45 min at 1,200 RPM in a benchtop centrifuge at room temperature [51, 52]. Infected macrophages were incubated for a further 3 h at 37°C before the addition of 300 μL of DMEM (10% human plasma) and incubating at 37°C for 7 days. DEAE dextran and spinoculation enhance virus infectivity by approximately 20-fold by increasing attachment [51] and entry [52]. Env^+^ pseudovirions are capable of only a single round of replication so that focus-forming units (FFU) were estimated 5–7 days post-infection by counting infected GFP+ macrophages by fluorescent microscopy.

## Supporting Information

S1 Table Datasets of amino acid fitness effects. The fitness effect, p value and classification (deleterious, wt-like and beneficial) of each amino acid substitution in P1 library (Excel file).
(XLSX)

S2 Table Beneficial mutations. (DOCX)

S3 Table The frequency of LN40 Env mutant amino acids on the HIV-1 database. (DOCX)

S4 Table The effect of LN40 mutations identified by EMPIRIC on Env structure and function. Neutralization assays were used to assess changes in Env structure and function.
(DOCX)

S5 Table N160 enhances the sensitivity of LN40 wt and mutant Envs to sCD4 and mab neutralization (fold change). (DOCX)

S6 Table N160 enhances the sensitivity of LN40 wt and mutant Envs to sCD4 and mab neutralization (IC50s). (DOCX)

S7 Table The effect of mutations identified by EMPIRIC on LN8 Env structure and function (fold change). Neutralization assays assessed changes in Env structure and function.
(DOCX)

S8 Table The effect of mutations identified by EMPIRIC on LN8 Env structure and function (IC50s). Neutralization assays assessed changes in Env structure and function.
(DOCX)

S9 Table The effect of mutations identified by EMPIRIC on Z1792M Env structure and function (fold change). (DOCX)

S10 Table The effect of mutations identified by EMPIRIC on Z1792M Env structure and function (IC50s). (DOCX)

S11 Table Neutralization sensitivity of LN40, LN8 and Z1792M wt, 375W, 377V and 380P Env+ pseudoviruses to potent CD4bs mabs VRC01 and 3BNC117 and gp120/gp41 junction mab, 8ANC195. (DOCX)

S12 Table Datasets of sequencing counts and frequency observations. Sequencing counts and frequency of each codon substitution in plasmid, P0, two experimental replicates of P1 libraries and *wt* plasmid (Excel file).
(XLSX)

S13 Table Datasets of codon fitness effects. The fitness effect of each codon substitution in two experimental replicates of P1 library (Excel file).
(XLSX)

S14 Table PCR primers used to introduce individual mutations into Env expression vectors. (DOCX)

S1 Fig Frequency of mutations in plasmid library, P0 and P1 viruses. The frequency of mutations at position 377 in plasmid library (A), P0 library (B), P1 library (C) and *wt* plasmid as noise level estimate (D). (E) The frequencies of mutants were strongly correlated in P0 library and in plasmid library. The data shown for residue 377 is representative of that for other residues.
(PPTX)

S2 Fig EMPIRIC protocol, depletion of stop codons and reproducibility between assays. (A) A cassette ligation strategy was used to introduce all 64 possible codons into each position of the CD4 binding loop region to form two libraries encompassing residues 361–370 and 371–380. (B) Depletion of stop codons and enrichment of *wt* synonyms (change in log2 frequency) in two experimental replicates of each library. Stop codons are shown in red and *wt* synonyms are shown in green. (C and D) Reproducibility of EMPIRIC measurements in HIV. Correlation in selection coefficient of ~600 point mutants at amino acid positions 361–370 (C) and 371–380 (D) in the Env gene following infection of PBMCs. Green spots in panels B and C represent codons synonymous with *wt* codons.
(PPTX)

S3 Fig The presence of epitopes for mab 17b and V2q mabs on LN40 and absence of V2q epitope on Z1792M gp120. (A) ELISA showing enhanced exposure of the 17b epitope on LN40 monomeric gp120 in the presence of increasing concentrations of sCD4. (B) Introduction of N160 into LN40 did not confer sensitivity to neutralization by V2q mabs, PG9, PG16, PGT145. (C) PG9 and PGT145 do not bind monomeric Z1792M gp120. ELISAs showing HIV+ human serum mix (QC sera), PG9 and PGT145 interactions with captured gp120.
(PPTX)

S4 Fig Soluble CD4 abrogates the trimer apex V2q epitope on LN8 S375W envelope. LN8 375W Env was expressed on 293T cells, and stained with PGT145 mab in the presence and absence of sCD4.
(PPTX)

S5 Fig Alternative models to explain how Env trimers may expose the CD4bs while retaining a closed TAD at the trimer apex. (A) Localized conformational changes expose CD4bs epitopes without impacting the TAD and V2q epitopes. (B) The Env trimer moves from a closed to open form and back again. V2q mabs capture the closed form, CD4bs mabs capture the open form.
(PPTX)

S6 Fig CD4 binding loop residues identified that control trimer conformation. Residues are depicted as spheres in different colors. The V1 (yellow), V2 (green) and V3 (orange) loops that form the trimer association domain (TAD) are shown at the trimer apex. Light yellow spheres show the NAG (N-acetyl glucosamine) of N160 (Cyan) on the top of the trimer. CD4 contact sites (2), are red in the cartoon structure. Structure is based on a side view of trimer (PDB 4NCO [12]). See also Movie at https://vimeo.com/165897330, password: Mama8).
(PPTX)

## References

[1] Wilen CB, Tilton JC, Doms RW. Molecular mechanisms of HIV entry. Adv Exp Med Biol. 2012;726:223–42. 10.1007/978-1-4614-0980-9_10 22297516

[2] Kwong PD, Wyatt R, Robinson J, Sweet RW, Sodroski J, Hendrickson WA. Structure of an HIV gp120 envelope glycoprotein in complex with the CD4 receptor and a neutralizing human antibody. Nature. 1998;393:648–59. 10.1038/31405 9641677 PMC5629912

[3] Madani N, Schon A, Princiotto AM, Lalonde JM, Courter JR, Soeta T, et al Small-molecule CD4 mimics interact with a highly conserved pocket on HIV-1 gp120. Structure. 2008;16:1689–701. 10.1016/j.str.2008.09.005 19000821 PMC2597202

[4] Haim H, Strack B, Kassa A, Madani N, Wang L, Courter JR, et al Contribution of intrinsic reactivity of the HIV-1 envelope glycoproteins to CD4-independent infection and global inhibitor sensitivity. PLoS Pathog. 2011;7:e1002101 10.1371/journal.ppat.1002101 21731494 PMC3121797

[5] Xiang SH, Kwong PD, Gupta R, Rizzuto CD, Casper DJ, Wyatt R, et al Mutagenic stabilization and/or disruption of a CD4-bound state reveals distinct conformations of the human immunodeficiency virus type 1 gp120 envelope glycoprotein. J Virol. 2002;76:9888–99. 10.1128/JVI.76.19.9888-9899.2002 12208966 PMC136507

[6] Peters PJ, Bhattacharya J, Hibbitts S, Dittmar MT, Simmons G, Bell J, et al Biological analysis of human immunodeficiency virus type 1 R5 envelopes amplified from brain and lymph node tissues of AIDS patients with neuropathology reveals two distinct tropism phenotypes and identifies envelopes in the brain that confer an enhanced tropism and fusigenicity for macrophages. J Virol. 2004;78:6915–26. 10.1128/JVI.78.13.6915-6926.2004 15194768 PMC421670

[7] Peters PJ, Duenas-Decamp MJ, Sullivan WM, Brown R, Ankghuambom C, Luzuriaga K, et al Variation in HIV-1 R5 macrophage-tropism correlates with sensitivity to reagents that block envelope: CD4 interactions but not with sensitivity to other entry inhibitors. Retrovirology. 2008;5:5 10.1186/1742-4690-5-5 18205925 PMC2268948

[8] Duenas-Decamp MJ, Peters PJ, Burton D, Clapham PR. Determinants flanking the CD4 binding loop modulate macrophage tropism of human immunodeficiency virus type 1 R5 envelopes. J Virol. 2009;83:2575–83. 10.1128/JVI.02133-08 19129457 PMC2648272

[9] Dunfee RL, Thomas ER, Gorry PR, Wang J, Taylor J, Kunstman K, et al The HIV Env variant N283 enhances macrophage tropism and is associated with brain infection and dementia. Proc Natl Acad Sci U S A. 2006;103:15160–5. 10.1073/pnas.0605513103 17015824 PMC1586182

[10] Musich T, Peters PJ, Duenas-Decamp MJ, Gonzalez-Perez MP, Robinson J, Zolla-Pazner S, et al A conserved determinant in the V1 loop of HIV-1 modulates the V3 loop to prime low CD4 use and macrophage infection. J Virol. 2011;85:2397–405. 10.1128/JVI.02187-10 21159865 PMC3067776

[11] Walter BL, Wehrly K, Swanstrom R, Platt E, Kabat D, Chesebro B. Role of low CD4 levels in the influence of human immunodeficiency virus type 1 envelope V1 and V2 regions on entry and spread in macrophages. J Virol. 2005;79:4828–37. 10.1128/JVI.79.8.4828-4837.2005 15795268 PMC1069537

[12] Julien JP, Cupo A, Sok D, Stanfield RL, Lyumkis D, Deller MC, et al Crystal structure of a soluble cleaved HIV-1 envelope trimer. Science. 2013;342:1477–83. 10.1126/science.1245625 24179159 PMC3886632

[13] Hietpas R, Roscoe B, Jiang L, Bolon DN. Fitness analyses of all possible point mutations for regions of genes in yeast. Nat Protoc. 2012;7:1382–96. 10.1038/nprot.2012.069 22722372 PMC3509169

[14] Hietpas RT, Bank C, Jensen JD, Bolon DN. Shifting fitness landscapes in response to altered environments. Evolution. 2013;67:3512–22. 10.1111/evo.12207 24299404 PMC3855258

[15] Hietpas RT, Jensen JD, Bolon DN. Experimental illumination of a fitness landscape. Proc Natl Acad Sci U S A. 2011;108:7896–901. 10.1073/pnas.1016024108 21464309 PMC3093508

[16] Jiang L, Mishra P, Hietpas RT, Zeldovich KB, Bolon DN. Latent effects of Hsp90 mutants revealed at reduced expression levels. PLoS Genet. 2013;9:e1003600 10.1371/journal.pgen.1003600 23825969 PMC3694843

[17] Wagenaar TR, Ma L, Roscoe B, Park SM, Bolon DN, Green MR. Resistance to vemurafenib resulting from a novel mutation in the BRAFV600E kinase domain. Pigment cell & melanoma research. 2014;27:124–33. 24112705 10.1111/pcmr.12171 PMC4260813

[18] Roscoe BP, Thayer KM, Zeldovich KB, Fushman D, Bolon DN. Analyses of the effects of all ubiquitin point mutants on yeast growth rate. J Mol Biol. 2013;425:1363–77. 10.1016/j.jmb.2013.01.032 23376099 PMC3615125

[19] Duenas-Decamp MJ, Clapham PR. HIV-1 gp120 determinants proximal to the CD4 binding site shift protective glycans that are targeted by monoclonal antibody, 2G12. J Virol. 2010;84: 9608–12. 10.1128/JVI.00185-10 20610714 PMC2937615

[20] Duenas-Decamp MJ, Peters P, Burton D, Clapham PR. Natural resistance of human immunodeficiency virus type 1 to the CD4bs antibody b12 conferred by a glycan and an arginine residue close to the CD4 binding loop. J Virol. 2008;82:5807–14. 10.1128/JVI.02585-07 18385254 PMC2395159

[21] Peters PJ, Sullivan WM, Duenas-Decamp MJ, Bhattacharya J, Ankghuambom C, Brown R, et al Non-macrophage-tropic human immunodeficiency virus type 1 R5 envelopes predominate in blood, lymph nodes, and semen: implications for transmission and pathogenesis. J Virol. 2006;80:6324–32. 10.1128/JVI.02328-05 16775320 PMC1488974

[22] Sturdevant CB, Joseph SB, Schnell G, Price RW, Swanstrom R, Spudich S. Compartmentalized replication of R5 T cell-tropic HIV-1 in the central nervous system early in the course of infection. PLoS Pathog. 2015;11:e1004720 10.1371/journal.ppat.1004720 25811757 PMC4374811

[23] Schnell G, Joseph S, Spudich S, Price RW, Swanstrom R. HIV-1 replication in the central nervous system occurs in two distinct cell types. PLoS Pathog. 2011;7:e1002286 10.1371/journal.ppat.1002286 22007152 PMC3188520

[24] Peters PJ, Gonzalez-Perez MP, Musich T, Moore Simas TA, Lin R, Morse AN, et al Infection of ectocervical tissue and universal targeting of T-cells mediated by primary non-macrophage-tropic and highly macrophage-tropic HIV-1 R5 envelopes. Retrovirology. 2015;12:48 10.1186/s12977-015-0176-2 26055104 PMC4459458

[25] Isaacman-Beck J, Hermann EA, Yi Y, Ratcliffe SJ, Mulenga J, Allen S, et al Heterosexual transmission of human immunodeficiency virus type 1 subtype C: Macrophage tropism, alternative coreceptor use, and the molecular anatomy of CCR5 utilization. J Virol. 2009;83:8208–20. 10.1128/JVI.00296-09 19515785 PMC2715751

[26] Salazar-Gonzalez JF, Salazar MG, Keele BF, Learn GH, Giorgi EE, Li H, et al Genetic identity, biological phenotype, and evolutionary pathways of transmitted/founder viruses in acute and early HIV-1 infection. J Exp Med. 2009;206:1273–89. 10.1084/jem.20090378 19487424 PMC2715054

[27] Joseph SB, Swanstrom R, Kashuba AD, Cohen MS. Bottlenecks in HIV-1 transmission: insights from the study of founder viruses. Nat Rev Microbiol. 2015;13:414–25. 10.1038/nrmicro3471 26052661 PMC4793885

[28] Meyerson JR, Tran EE, Kuybeda O, Chen W, Dimitrov DS, Gorlani A, et al Molecular structures of trimeric HIV-1 Env in complex with small antibody derivatives. Proc Natl Acad Sci U S A. 2013;110:513–8. 10.1073/pnas.1214810110 23267106 PMC3545814

[29] Tran EE, Borgnia MJ, Kuybeda O, Schauder DM, Bartesaghi A, Frank GA, et al Structural mechanism of trimeric HIV-1 envelope glycoprotein activation. PLoS Pathog. 2012;8:e1002797 10.1371/journal.ppat.1002797 22807678 PMC3395603

[30] O'Connell O, Repik A, Reeves JD, Gonzalez-Perez MP, Quitadamo B, Anton ED, et al Efficiency of bridging-sheet recruitment explains HIV-1 R5 envelope glycoprotein sensitivity to soluble CD4 and macrophage tropism. J Virol. 2013;87:187–98. 10.1128/JVI.01834-12 23055568 PMC3536387

[31] Dey B, Pancera M, Svehla K, Shu Y, Xiang SH, Vainshtein J, et al Characterization of human immunodeficiency virus type 1 monomeric and trimeric gp120 glycoproteins stabilized in the CD4-bound state: antigenicity, biophysics, and immunogenicity. J Virol. 2007;81:5579–93. 10.1128/JVI.02500-06 17360741 PMC1900256

[32] Binley JM, Wrin T, Korber B, Zwick MB, Wang M, Chappey C, et al Comprehensive cross-clade neutralization analysis of a panel of anti-human immunodeficiency virus type 1 monoclonal antibodies. J Virol. 2004;78:13232–52. 10.1128/JVI.78.23.13232-13252.2004 15542675 PMC524984

[33] Hoffman TL, LaBranche CC, Zhang W, Canziani G, Robinson J, Chaiken I, et al Stable exposure of the coreceptor-binding site in a CD4-independent HIV- 1 envelope protein. Proc Natl Acad Sci U S A. 1999;96:6359–64. 10339592 10.1073/pnas.96.11.6359 PMC26886

[34] Boyd DF, Peterson D, Haggarty BS, Jordan AP, Hogan MJ, Goo L, et al Mutations in HIV-1 envelope that enhance entry with the macaque CD4 receptor alter antibody recognition by disrupting quaternary interactions within the trimer. J Virol. 2015;89:894–907. 10.1128/JVI.02680-14 25378497 PMC4300673

[35] Murphy MK, Yue L, Pan R, Boliar S, Sethi A, Tian J, et al Viral escape from neutralizing antibodies in early subtype A HIV-1 infection drives an increase in autologous neutralization breadth. PLoS Pathog. 2013;9:e1003173 10.1371/journal.ppat.1003173 23468623 PMC3585129

[36] Walker LM, Phogat SK, Chan-Hui PY, Wagner D, Phung P, Goss JL, et al Broad and Potent Neutralizing Antibodies from an African Donor Reveal a New HIV-1 Vaccine Target. Science. 2009;326:285–9. 10.1126/science.1178746 19729618 PMC3335270

[37] Munro JB, Gorman J, Ma X, Zhou Z, Arthos J, Burton DR, et al Conformational dynamics of single HIV-1 envelope trimers on the surface of native virions. Science. 2014;346(6210):759–63. 10.1126/science.1254426 25298114 PMC4304640

[38] Zwick MB, Burton DR. HIV-1 neutralization: mechanisms and relevance to vaccine design. Curr HIV Res. 2007;5:608–24. 18045117 10.2174/157016207782418443

[39] Gonzalez-Perez MP, O'Connell O, Lin R, Sullivan WM, Bell J, Simmonds P, et al Independent evolution of macrophage-tropism and increased charge between HIV-1 R5 envelopes present in brain and immune tissue. Retrovirology. 2012;9:20 10.1186/1742-4690-9-20 22420378 PMC3362761

[40] Thomas ER, Dunfee RL, Stanton J, Bogdan D, Taylor J, Kunstman K, et al Macrophage entry mediated by HIV Envs from brain and lymphoid tissues is determined by the capacity to use low CD4 levels and overall efficiency of fusion. Virology. 2007;360:105–19. 10.1016/j.virol.2006.09.036 17084877 PMC1890014

[41] Li Y, O'Dell S, Walker LM, Wu X, Guenaga J, Feng Y, et al Mechanism of neutralization by the broadly neutralizing HIV-1 monoclonal antibody VRC01. J Virol. 2011;85:8954–67. 10.1128/JVI.00754-11 21715490 PMC3165784

[42] Scheid JF, Mouquet H, Ueberheide B, Diskin R, Klein F, Oliveira TY, et al Sequence and structural convergence of broad and potent HIV antibodies that mimic CD4 binding. Science. 2011;333:1633–7. 10.1126/science.1207227 21764753 PMC3351836

[43] Scharf L, Wang H, Gao H, Chen S, McDowall AW, Bjorkman PJ. Broadly Neutralizing Antibody 8ANC195 Recognizes Closed and Open States of HIV-1 Env. Cell. 2015;162:1379–90. 10.1016/j.cell.2015.08.035 26359989 PMC4587768

[44] Li H, Wang S, Kong R, Ding W, Lee FH, Parker Z, et al Envelope residue 375 substitutions in simian-human immunodeficiency viruses enhance CD4 binding and replication in rhesus macaques. Proc Natl Acad Sci U S A. 2016;113:E3413–22. 10.1073/pnas.1606636113 27247400 PMC4914158

[45] Li MZ, Elledge SJ. SLIC: a method for sequence- and ligation-independent cloning. Methods Mol Biol. 2012;852:51–9. 10.1007/978-1-61779-564-0_5 22328425

[46] Wei X, Decker JM, Liu H, Zhang Z, Arani RB, Kilby JM, et al Emergence of resistant human immunodeficiency virus type 1 in patients receiving fusion inhibitor (T-20) monotherapy. Antimicrob Agents Chemother. 2002;46:1896–905. 10.1128/AAC.46.6.1896-1905.2002 12019106 PMC127242

[47] Musich T, O'Connell O, Gonzalez-Perez MP, Derdeyn CA, Peters PJ, Clapham PR. HIV-1 non-macrophage-tropic R5 envelope glycoproteins are not more tropic for entry into primary CD4+ T-cells than envelopes highly adapted for macrophages. Retrovirology. 2015;12:25 10.1186/s12977-015-0141-0 25809903 PMC4373511

[48] Guo W, Cleveland B, Davenport TM, Lee KK, Hu SL. Purification of recombinant vaccinia virus-expressed monomeric HIV-1 gp120 to apparent homogeneity. Protein Expr Purif. 2013;90:34–9. 10.1016/j.pep.2013.04.009 23665667 PMC3718289

[49] Moore JP. Simple methods for monitoring HIV-1 and HIV-2 gp120 binding to soluble CD4 by enzyme-linked immunosorbent assay: HIV-2 has a 25-fold lower affinity than HIV-1 for soluble CD4. Aids. 1990;4:297–305. 2190604 10.1097/00002030-199004000-00003

[50] Ringe RP, Sanders RW, Yasmeen A, Kim HJ, Lee JH, Cupo A, et al Cleavage strongly influences whether soluble HIV-1 envelope glycoprotein trimers adopt a native-like conformation. Proc Natl Acad Sci U S A. 2013;110:18256–61. 10.1073/pnas.1314351110 24145402 PMC3831437

[51] Guo J, Wang W, Yu D, Wu Y. Spinoculation triggers dynamic actin and cofilin activity that facilitates HIV-1 infection of transformed and resting CD4 T cells. J Virol. 2011;85:9824–33. 10.1128/JVI.05170-11 21795326 PMC3196392

[52] O'Doherty U, Swiggard WJ, Malim MH. Human immunodeficiency virus type 1 spinoculation enhances infection through virus binding. J Virol. 2000;74:10074–80. 11024136 10.1128/jvi.74.21.10074-10080.2000 PMC102046

