# Supplementary material for: Saturation Mutagenesis of the HIV-1 Envelope CD4 Binding Loop Reveals Residues Controlling Distinct Trimer Conformations
Source: PLoS Pathog. 2016 Nov 7;12(11):e1005988. doi: 10.1371/journal.ppat.1005988 (PMC5098743; doi:10.1371/journal.ppat.1005988)
Supplement: S2 Table — (DOCX) [file ppat.1005988.s002.docx]

**S2 Table. Beneficial mutations.**

Mutant Fitness effect

R373E 0.36

R373K 0.34

R373N 0.24

R373Q 0.43

S375F 0.63

S375H 0.63

S375T 0.44

S375W 0.36

S375Y 0.64

N377L 0.16

N377T 0.18

N377V 0.49

G380P 0.27
