# Supplementary material for: Saturation Mutagenesis of the HIV-1 Envelope CD4 Binding Loop Reveals Residues Controlling Distinct Trimer Conformations
Source: PLoS Pathog. 2016 Nov 7;12(11):e1005988. doi: 10.1371/journal.ppat.1005988 (PMC5098743; doi:10.1371/journal.ppat.1005988)
Supplement: S3 Table — (DOCX) [file ppat.1005988.s003.docx]

**S3 Table. The frequency of LN40 Env mutant amino acids on the HIV-1 database.**

| Residue | Mutant amino acid | % of strains with mutant amino acid^1^ | Residue | Mutant amino acid | % of strains with mutant amino acid |
| --- | --- | --- | --- | --- | --- |
| 361 | F *wt* | 98.89 | 371 | R *wt* | 0.02 |
|  | I | 0.02 |  | V | 12.19 |
|  | L | 0.32 |  |  |  |
|  | Y | 0.3 | 373 | R *wt* | 0.93 |
|  |  |  |  | K | 0.61 |
| 362 | N *wt* | 38.19 |  | M | 43.78 |
|  | D | 2.57 |  | Q | 0.30 |
|  | E | 7.19 |  | E | 0.07 |
|  | K | 15.97 |  | N | 0.07 |
|  | S | 1.21 |  |  |  |
|  | T | 7.73 | 375 | S *wt* | 75.14 |
|  | A | 9.03 |  | H | 11.19 |
|  |  |  |  | T | 8.73 |
| 363 | Q *wt* | 24.27 |  | F | 0.11 |
|  | D | 0.09 |  | W | 0.00 |
|  | E | 0.93 |  | Y | 0.05 |
|  | G | 0.34 |  |  |  |
|  | H | 6.30 | 377 | N *wt* | 95.57 |
|  |  |  |  | V | 0.20 |
| 365 | S *wt* | 87.20 |  |  |  |
|  | A | 6.53 | 380 | G *wt* | 99.61 |
|  | V | 0.77 |  | A | 0.00 |
|  |  |  |  | V | 0.20 |
| 369 | P *wt* | 44.23 |  |  |  |
|  | A | 0.14 |  |  |  |
|  | C | 0.00 |  |  |  |
|  | D | 0.00 |  |  |  |
|  | E | 0.00 |  |  |  |
| 1. % of Envs among the major subtypes (A, B, C, D, F, G, CRF01 and CRF02) on the HIV-1 sequence database (<http://www.hiv.lanl.gov/content/sequence/HIV/mainpage.html)>. | | | | | |
