# Supplementary material for: Saturation Mutagenesis of the HIV-1 Envelope CD4 Binding Loop Reveals Residues Controlling Distinct Trimer Conformations
Source: PLoS Pathog. 2016 Nov 7;12(11):e1005988. doi: 10.1371/journal.ppat.1005988 (PMC5098743; doi:10.1371/journal.ppat.1005988)
Supplement: S4 Table — Neutralization assays were used to assess changes in Env structure and function. (DOCX) [file ppat.1005988.s004.docx]

| **S4 Table. The effect of LN40 mutations identified by EMPIRIC on Env structure and function.**  Neutralization assays were used to assess changes in Env structure and function | | | | | | | | |
| --- | --- | --- | --- | --- | --- | --- | --- | --- |
| LN40 Env *wt* and mutants | | sCD4 | 447-52D  *-V3 crown* | b6  *-CD4bs* | b12  *-CD4bs* | 17b  *-CD4i* | PGT128 | 2G12  *-glycans* |
|  |  | IC50s (μg/ml) | | | | | | |
| LN40 *wt* | | >50 | 40 | >50 | 34.9 | >50 | 0.008 | 5.2 |
| 361 | F361I | 49.1 | 32.8 | >50 | >50 | nt | 0.007 | 3.1 |
|  | F361L | >50 | 34.6 | >50 | 40.7 | nt | 0.007 | 3.5 |
|  | F361Y | 35.8 | 30 | >50 | >50 | nt | 0.008 | 1.9 |
| 362 | N362D | 39.9 | 33.8 | >50 | 29.6 | nt | 0.008 | 9.3 |
|  | N362E | >50 | 29.2 | >50 | >50 | nt | 0.008 | 3.5 |
|  | N362K | >50 | 21.6 | >50 | >50 | nt | 0.007 | 1.1 |
|  | N362S | >50 | >50 | >50 | >50 | nt | 0.006 | 1.9 |
|  | N362T | >50 | 20 | >50 | >50 | nt | 0.008 | 2.0 |
|  | N362A | >50 | >50 | >50 | >50 | nt | 0.007 | 1.9 |
| 363 | Q363D | 36.8 | 17.8 | >50 | 4.5 | nt | 0.008 | 11.1 |
|  | Q363E | 34.7 | 18.1 | >50 | 2.5 | nt | 0.007 | 10.7 |
|  | Q363G | >50 | 29.4 | >50 | >50 | nt | 0.007 | 6.3 |
|  | Q363H | 27.2 | 14.5 | >50 | >50 | nt | 0.008 | 6.1 |
| 365 | S365A | 32.1 | 21.7 | >50 | >50 | nt | 0.009 | 5.0 |
|  | S365V | 44.6 | 28.5 | >50 | >50 | >50 | 0.008 | 5.2 |
| 369 | P369A | >50 | >50 | >50 | >50 | nt | nt | 9.0 |
|  | P369C | >50 | >50 | >50 | >50 | nt | nt | 5.1 |
|  | P369D | >50 | 26 | >50 | >50 | nt | nt | 14 |
|  | P369E | >50 | 29 | >50 | >50 | nt | nt | 10 |
| 371 | R371V | >50 | >50 | >50 | 34.5 | nt | 0.01 | 5.0 |
| 373 | R373K | >50 | 35.2 | >50 | 2.8 | nt | 0.009 | 8.2 |
|  | R373M | >50 | >50 | >50 | 1.1 | nt | 0.009 | 7.5 |
|  | R373Q | 44.2 | 25.2 | >50 | 1.5 | nt | 0.012 | 14.9 |
|  | R373E | 23.9 | 2.4 | >50 | 2.0 | >50 | 0.012 | 26.4 |
|  | R373N | 43.9 | 12.9 | >50 | 3.4 | >50 | 0.01 | 9.9 |
| 375 | S375H | 15.3 | >50 | >50 | <50 | nt | 0.014 | 15.9 |
|  | S375T | 38.2 | >50 | >50 | 34.0 | nt | 0.009 | 8.4 |
|  | S375F | 7.6 | >50 | >50 | >50 | nt | 0.013 | 21.3 |
|  | S375W | 8.7 | >50 | >50 | 42.8 | >50 | 0.014 | 32.4 |
|  | S375Y | 5.9 | >50 | >50 | 45.4 | >50 | 0.01 | 30.1 |
| 377 | N377V | 19.0 | 1.3 | >50 | 41.2 | >50 | 0.01 | 12.3 |
| 380 | G380A | 32.0 | <0.2 | 33.5 | 26.9 | >50 | 0.01 | 11.4 |
|  | G380P | 21.7 | <0.2 | 15.3 | 25.2 | >50 | 0.02 | 26.4 |
| nt; not tested  green, >10<25; yellow, >1<10, red, <1. | | | | | | | | |
