# Supplementary material for: Saturation Mutagenesis of the HIV-1 Envelope CD4 Binding Loop Reveals Residues Controlling Distinct Trimer Conformations
Source: PLoS Pathog. 2016 Nov 7;12(11):e1005988. doi: 10.1371/journal.ppat.1005988 (PMC5098743; doi:10.1371/journal.ppat.1005988)
Supplement: S5 Table — (DOCX) [file ppat.1005988.s005.docx]

| Mab, inhibitor | Env | Fold change in IC50s from LN40wt  +/- N160 | |
| --- | --- | --- | --- |
|  |  | - | + |
| sCD4 | *wt* | 1.00 | 2.36 |
|  | 373E | 1.85 | 5.95 |
|  | 375W | 5.75 | 10.87 |
|  | 377V | 2.63 | 23.81 |
|  | 380P | 2.25 | 8.20 |
| 447-52D | *wt* | 1.54 | 10.64 |
|  | 373E | 20.83 | 250.00 |
|  | 375W | 1.00 | 2.07 |
|  | 377V | 45.45 | 6250.00 |
|  | 380P | 3571.43 | 12500.00 |
| b6 | *wt* | 1.00 | 1.00 |
|  | 373E | 1.00 | 2.56 |
|  | 375W | 1.00 | 1.00 |
|  | 377V | 1.00 | 2.67 |
|  | 380P | 3.27 | 11.63 |
| b12 | *wt* | 1.73 | 1.09 |
|  | 373E | 25.00 | 35.71 |
|  | 375W | 1.00 | 1.00 |
|  | 377V | 1.00 | 1.00 |
|  | 380P | 1.00 | 1.00 |
| green, >2,<4-fold; yellow, >4<100-fold; red, >100-fold differences. | | | |

**S5 Table. N160 enhances the sensitivity of LN40 wt and mutant Envs to sCD4 and mab neutralization.**
