# Supplementary material for: Saturation Mutagenesis of the HIV-1 Envelope CD4 Binding Loop Reveals Residues Controlling Distinct Trimer Conformations
Source: PLoS Pathog. 2016 Nov 7;12(11):e1005988. doi: 10.1371/journal.ppat.1005988 (PMC5098743; doi:10.1371/journal.ppat.1005988)
Supplement: S6 Table — (DOCX) [file ppat.1005988.s006.docx]

**S6 Table. N160 enhances the sensitivity of LN40 wt and mutant Envs to sCD4 and mab neutralization.**

| Mab, inhibitor | Env | IC50s  +/- N160 | |
| --- | --- | --- | --- |
|  |  | - | + |
| sCD4 | *wt* | >50 | 21.2 |
|  | 373E | 27.0 | 8.4 |
|  | 375W | 8.7 | 4.6 |
|  | 377V | 19.0 | 2.1 |
|  | 380P | 22.2 | 6.1 |
| 447-52D | *wt* | 32.4 | 4.7 |
|  | 373E | 2.4 | <0.2 |
|  | 375W | >50 | 24.2 |
|  | 377V | 1.1 | 0.008 |
|  | 380P | 0.014 | 0.004 |
| b6 | *wt* | >50 | >50 |
|  | 373E | >50 | 19.5 |
|  | 375W | >50 | >50 |
|  | 377V | >50 | 18.7 |
|  | 380P | 15.3 | 4.3 |
| b12 | *wt* | 28.9 | 45.9 |
|  | 373E | 2.0 | 1.4 |
|  | 375W | >50 | >50 |
|  | 377V | >50 | >50 |
|  | 380P | >50 | >50 |
| green, >10<25; yellow, >1<10, red, <1. | | | |
