# Supplementary material for: Saturation Mutagenesis of the HIV-1 Envelope CD4 Binding Loop Reveals Residues Controlling Distinct Trimer Conformations
Source: PLoS Pathog. 2016 Nov 7;12(11):e1005988. doi: 10.1371/journal.ppat.1005988 (PMC5098743; doi:10.1371/journal.ppat.1005988)
Supplement: S7 Table — Neutralization assays assessed changes in Env structure and function. (DOCX) [file ppat.1005988.s007.docx]

| **S7 Table. The effect of mutations identified by EMPIRIC on LN8 Env structure and function.** Neutralization assays assessed changes in Env structure and function | | | | | | | | | |  |
| --- | --- | --- | --- | --- | --- | --- | --- | --- | --- | --- |
| LN8 Env  *wt* and mutants | | sCD4 | 447-52D  *-V3 crown* | b6  *-CD4bs* | b12  *-CD4bs* | 2G12  *-glycans* | PGT128  *-V3 glycans* | PG9  *-V2 N160* | PGT145  *-V2 N160* | |
|  |  | Fold change in IC50s | | | | | | |  | |
| LN8  *wt* | | 1.00 | 1.00 | 1.00 | 1.00 | 1.00 | 1.00 | 1.00 | 1.00 | |
| 362 | N362D | 1.30 | 1.00 | 1.00 | 10.00 | 0.12 | 0.58 | 0.89 | 0.86 | |
| 363 | Q363D | 1.18 | 1.00 | 1.00 | 2.04 | 0.28 | 0.70 | 1.06 | 1.50 | |
| 365 | S365A | 1.60 | 1.00 | 1.00 | 0.59 | 0.93 | 0.68 | 1.31 | 1.00 | |
|  | S365V | 5.09 | 1.00 | 1.00 | 1.07 | 1.71 | 1.27 | 3.40 | 2.00 | |
| 373 | M373E | 1.09 | 1.34 | 1.00 | 0.57 | 0.88 | 1.73 | 1.55 | 1.50 | |
|  | M373N | 1.18 | 1.00 | 1.00 | 0.57 | 0.90 | 1.58 | 0.77 | 1.20 | |
| 375 | S375H | 3.31 | 1.00 | 1.00 | 0.57 | 0.20 | 0.70 | 0.17 | 0.67 | |
|  | S375W | 11.97 | 1.00 | 1.00 | 0.57 | 0.17 | 0.41 | 0.03 | 0.55 | |
| 377 | N377V | 4.15 | 1.36 | 1.00 | 0.95 | 0.92 | 1.19 | 0.77 | 0.86 | |
| 380 | G380A | 1.69 | 13.89 | 1.00 | 13.67 | 0.84 | 0.95 | 1.06 | 0.86 | |
|  | G380P | 53.55 | 250.00 | 250.00 | 143.50 | 3.33 | 1.00 | 0.04 | 0.38 | |
| green, >2,<4-fold; yellow, >4<100-fold; red, >100-fold differences. | | | | | | | | | | |
