# Supplementary material for: Saturation Mutagenesis of the HIV-1 Envelope CD4 Binding Loop Reveals Residues Controlling Distinct Trimer Conformations
Source: PLoS Pathog. 2016 Nov 7;12(11):e1005988. doi: 10.1371/journal.ppat.1005988 (PMC5098743; doi:10.1371/journal.ppat.1005988)
Supplement: S8 Table — Neutralization assays assessed changes in Env structure and function. (DOCX) [file ppat.1005988.s008.docx]

| **S8 Table. The effect of mutations identified by EMPIRIC on LN8 Env structure and function.** Neutralization assays assessed changes in Env structure and function | | | | | | | | | |
| --- | --- | --- | --- | --- | --- | --- | --- | --- | --- |
| LN8 Env  *wt* and mutants | | sCD4 | 447-52D  *-V3 crown* | b6  *-CD4bs* | b12  *-CD4bs* | 2G12  *-glycans* | PGT128  *-V3 glycans* | PG9  *-V2 N160* | PGT145  *-V2 N160* |
|  |  | IC50s (μg/ml) | | | | | | |  |
| LN8  *wt* | | 40.7 | >50 | >50 | 28.7 | 6.16 | 0.019 | 0.17 | 0.0006 |
| 362 | N362D | 31.4 | >50 | >50 | 2.87 | >50 | 0.033 | 0.19 | 0.0007 |
| 363 | Q363D | 34.5 | >50 | >50 | 14.1 | 21.8 | 0.027 | 0.16 | 0.0004 |
| 365 | S365A | 25.4 | >50 | >50 | 48.5 | 6.59 | 0.028 | 0.13 | 0.0006 |
|  | S365V | 8.0* | >50 | >50 | 26.9 | 3.61 | 0.015 | 0.05 | 0.0003 |
| 373 | M373E | 37.5 | 37.4 | >50 | >50 | 6.98 | 0.011 | 0.11 | 0.0004 |
|  | M373N | 34.6 | >50 | >50 | >50 | 6.87 | 0.012 | 0.22 | 0.0005 |
| 375 | S375H | 12.3 | >50 | >50 | >50 | 30.8 | 0.027 | 1.0 | 0.0009 |
|  | S375W | 3.4 | >50 | >50 | >50 | 35.3 | 0.046 | 5.3 | 0.0011 |
| 377 | N377V | 9.8 | 36.7 | >50 | 30.1 | 6.71 | 0.016 | 0.22 | 0.0007 |
| 380 | G380A | 24.1 | 3.6 | >50 | 2.1 | 7.36 | 0.02 | 0.16 | 0.0007 |
|  | G380P | 0.76 | <0.2 | 0.2 | <0.2 | 1.85 | 0.019 | 4.3 | 0.0016 |
| sCD4, 447-52D, b6, b12, 2G12: green, >10<25; yellow, >1<10, red, <1.  PGT128: red, <0.1. PG9: yellow, >1; red, <1. PGT145: yellow, >0.001; red, <0.001.  *50% neutralization at 8.0 μg/ml. However, neutralization curves were erratic and neutralization incomplete, reaching a maximum of 75-85% at 50 μg/ml. | | | | | | | | | |
