# Supplementary material for: Saturation Mutagenesis of the HIV-1 Envelope CD4 Binding Loop Reveals Residues Controlling Distinct Trimer Conformations
Source: PLoS Pathog. 2016 Nov 7;12(11):e1005988. doi: 10.1371/journal.ppat.1005988 (PMC5098743; doi:10.1371/journal.ppat.1005988)
Supplement: S9 Table — (DOCX) [file ppat.1005988.s009.docx]

**S9 Table. The effect of mutations identified by EMPIRIC on Z1792M Env structure and function.**

| Z1792M Env *wt* and mutants | | sCD4 | b6  *-CD4bs* | PG9  *-V2 N160* | PGT145  *-V2 N160* |
| --- | --- | --- | --- | --- | --- |
|  |  | Fold change in IC50s (μg/ml) | | | |
| Z1792M *wt* | | 1.00 | 1.00 | 1.00 | 1.00 |
| 362 | E362D | 1.13 | 1.00 | 0.70 | 1.03 |
| 363 | E363D | 1.13 | 1.00 | 0.75 | 0.48 |
| 364 | H364S | 1.13 | 1.00 | 0.90 | 0.52 |
| 365 | S365A | 1.13 | 1.00 | 0.70 | 1.10 |
|  | S365V | 1.05 | 1.00 | 0.15 | 0.02 |
| 369 | L369P | 0.27 | 1.00 | 2.05 | 2.72 |
| 373 | T373E | 0.98 | 1.00 | 0.40 | 0.10 |
|  | T373M | 0.43 | 1.00 | 1.60 | 2.07 |
|  | T373K | 0.93 | 1.00 | 0.60 | 1.86 |
|  | T373Q | 0.98 | 1.00 | 1.00 | 1.03 |
| 375 | S375W | 0.01 | 1.00 | 13.00 | 13.79 |
|  | S375Y | 0.03 | 1.00 | 7.50 | 6.52 |
|  | S375F | 0.05 | 1.00 | 2.30 | 1.90 |
|  | S375H | 0.06 | 1.00 | 6.00 | 4.41 |
|  | S375T | 0.59 | 1.00 | 1.10 | 3.07 |
| 377 | N377V | 0.31 | 1.00 | 1.00 | 6.55 |
|  | N377L | 0.34 | 1.00 | 1.00 | 4.55 |
|  | N377T | 1.03 | 1.00 | 0.80 | 2.34 |
| 380 | G380A | 0.80 | 1.00 | 0.60 | 2.21 |
|  | G380P | 0.16 | 0.19 | 1.60 | 13.79 |
| green, >2,<4-fold; yellow, >4<100-fold; red, >100-fold differences. | | | | | |
