# Supplementary material for: Saturation Mutagenesis of the HIV-1 Envelope CD4 Binding Loop Reveals Residues Controlling Distinct Trimer Conformations
Source: PLoS Pathog. 2016 Nov 7;12(11):e1005988. doi: 10.1371/journal.ppat.1005988 (PMC5098743; doi:10.1371/journal.ppat.1005988)
Supplement: S10 Table — (DOCX) [file ppat.1005988.s010.docx]

**S10 Table. The effect of mutations identified by EMPIRIC on Z1792M Env structure and function.**

| Z1792M Env *wt* and mutants | | sCD4 | b6  *-CD4bs* | PG9  *-V2 N160* | PGT145  *-V2 N160* |
| --- | --- | --- | --- | --- | --- |
|  |  | IC50s (μg/ml) | | | |
| Z1792M *wt* | | 44.2 | >50 | 0.20 | 0.29 |
| 362 | E362D | >50 | >50 | 0.14 | 0.3 |
| 363 | E363D | >50 | >50 | 0.15 | 0.14 |
| 364 | H364S | >50 | >50 | 0.18 | 0.15 |
| 365 | S365A | >50 | >50 | 0.14 | 0.32 |
|  | S365V | 46.3 | >50 | 0.03 | 0.005 |
| 369 | L369P | 12.1 | >50 | 0.41 | 0.79 |
| 373 | T373E | 43.2 | >50 | 0.08 | 0.03 |
|  | T373M | 19.1 | >50 | 0.32 | 0.6 |
|  | T373K | 41.1 | >50 | 0.12 | 0.54 |
|  | T373Q | 43.1 | >50 | 0.20 | 0.3 |
| 375 | S375W | 0.57 | >50 | 2.6 | >4.0 |
|  | S375Y | 1.2 | >50 | 1.5 | 1.89 |
|  | S375F | 2.0 | >50 | 0.46 | 0.55 |
|  | S375H | 2.5 | >50 | 1.2 | 1.28 |
|  | S375T | 26.0 | >50 | 0.22 | 0.89 |
| 377 | N377V | 13.5 | >50 | 0.20 | 1.9 |
|  | N377L | 15.0 | >50 | 0.20 | 1.32 |
|  | N377T | 45.4 | >50 | 0.16 | 0.68 |
| 380 | G380A | 35.2 | >50 | 0.12 | 0.64 |
|  | G380P | 7.2 | 9.5 | 0.32 | >4.0 |
| sCD4, b6, b12: green, >10<50; yellow, >1<10, red, <1.  PG9, PGT145: yellow, >1; beige, >0.1<1.0; red, <0.1. | | | | | |
