# Supplementary material for: Saturation Mutagenesis of the HIV-1 Envelope CD4 Binding Loop Reveals Residues Controlling Distinct Trimer Conformations
Source: PLoS Pathog. 2016 Nov 7;12(11):e1005988. doi: 10.1371/journal.ppat.1005988 (PMC5098743; doi:10.1371/journal.ppat.1005988)
Supplement: S11 Table — (DOCX) [file ppat.1005988.s011.docx]

**S11 Table. Neutralization sensitivity of LN40, LN8 and Z1792M wt, 375W, 377V and 380P Env+ pseudoviruses to potent CD4bs mabs VRC01 and 3BNC117 and gp120/gp41 junction mab, 8ANC195.**

| Env | | IC50 μg/ml | | |
| --- | --- | --- | --- | --- |
|  |  | VRC01 | 3BNC117 | 8ANC195 |
| LN40 | wt | 0.135 | 0.02 | >50 |
|  | 375W | 0.70 | 0.07 | >50 |
|  | 377V | 0.46 | 0.05 | >50 |
|  | 380P | 0.31 | 0.05 | >50 |
|  |  |  |  |  |
| LN8 | wt | 0.33 | 0.04 | >10 |
|  | 375W | 0.97 | 0.1 | >10 |
|  | 377V | 0.4 | 0.058 | >10 |
|  | 380P | 0.62 | 0.09 | >10 |
|  |  |  |  |  |
| Z1792M | wt | >4 | >10 | 0.04 |
|  | 375W | >4 | >10 | 0.1 |
|  | 377V | >4 | >10 | 0.058 |
|  | 380P | >4 | >10 | 0.095 |

| Env | | fold change | | |
| --- | --- | --- | --- | --- |
|  |  | VRC01 | 3BNC117 | 8ANC195 |
| LN40 | wt | 1 | 1 |  |
|  | 375W | 5.19 | 3.5 |  |
|  | 377V | 3.41 | 2.5 |  |
|  | 380P | 2.3 | 2.5 |  |
|  |  |  |  |  |
| LN8 | wt | 1 | 1 |  |
|  | 375W | 2.93 | 2.5 |  |
|  | 377V | 1.21 | 1.45 |  |
|  | 380P | 1.88 | 2.25 |  |
|  |  |  |  |  |
| Z1792M | wt |  |  | 1 |
|  | 375W |  |  | 2.5 |
|  | 377V |  |  | 1.45 |
|  | 380P |  |  | 2.38 |
