# Supplementary material for: Saturation Mutagenesis of the HIV-1 Envelope CD4 Binding Loop Reveals Residues Controlling Distinct Trimer Conformations
Source: PLoS Pathog. 2016 Nov 7;12(11):e1005988. doi: 10.1371/journal.ppat.1005988 (PMC5098743; doi:10.1371/journal.ppat.1005988)
Supplement: S14 Table — (DOCX) [file ppat.1005988.s014.docx]

**S14 Table. PCR primers used to introduce individual mutations into Env expression vectors.**

| Primer Set | Primer nomenclature | Primer |
| --- | --- | --- |
| Set 1 | ln40_upKpnF | GGGTCACAGTCTATTATGGG |
|  | ln40_downKpnR | GTAAGTCATTGGTCTTAAAG |
| Set 2 | LN8_373E_F | CCAGAAATTGTAgagCACAGTTTTAAT |
|  | LN8_373E_R | ATTAAAACTGTGctcTACAATTTCTGG |
|  | LN8_373N_F | CCAGAAATTGTAaatCACAGTTTTAAT |
|  | LN8_373N_R | ATTAAAACTGTGattTACAATTTCTGG |
|  | LN8_375H_F | ATTGTAATGCACcacTTTAATTGTGGA |
|  | LN8_375H_R | TCCACAATTAAAgtgGTGCATTACAAT |
|  | LN8_375W_F | ATTGTAATGCACtggTTTAATTGTGGA |
|  | LN8_375W_R | TCCACAATTAAAccaGTGCATTACAAT |
|  | LN8_377V_F | ATGCACAGTTTTgtcTGTGGAGGGGAA |
|  | LN8_377V_R | TTCCCCTCCACAgacAAAACTGTGCAT |
|  | LN8_380P_F | TTTAATTGTGGAccgGAATTTTTCTAC |
|  | LN8_380P_R | GTAGAAAAATTCcggTCCACAATTAAA |
|  | LN8_380A_F | TTTAATTGTGGAgctGAATTTTTCTAC |
|  | LN8_380A_R | GTAGAAAAATTCagcTCCACAATTAAA |
