# Supplementary figures and images for: Saturation Mutagenesis of the HIV-1 Envelope CD4 Binding Loop Reveals Residues Controlling Distinct Trimer Conformations
Source: PLoS Pathog. 2016 Nov 7;12(11):e1005988. doi: 10.1371/journal.ppat.1005988 (PMC5098743; doi:10.1371/journal.ppat.1005988)

## Slide 1
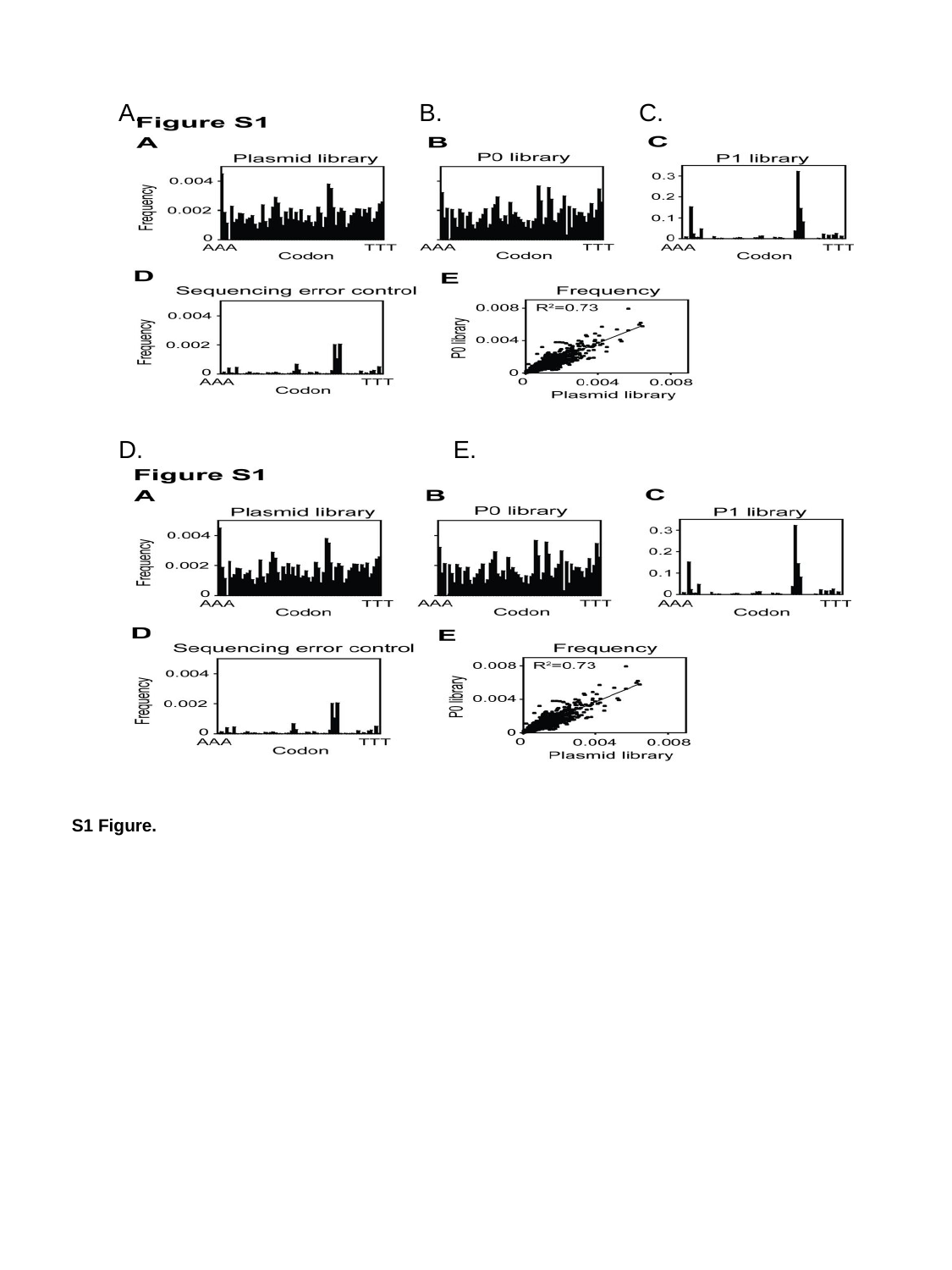

A.
B.
C.
D.
E.
S1 Figure.

Supplement: S1 Fig — The frequency of mutations at position 377 in plasmid library (A), P0 library (B), P1 library (C) and wt plasmid as noise level estimate (D). (E) The frequencies of mutants were strongly correlated in P0 library and in plasmid library. The data shown for residue 377 is representative of that for other residues. (PPTX) [file ppat.1005988.s015.pptx]

## Slide 1
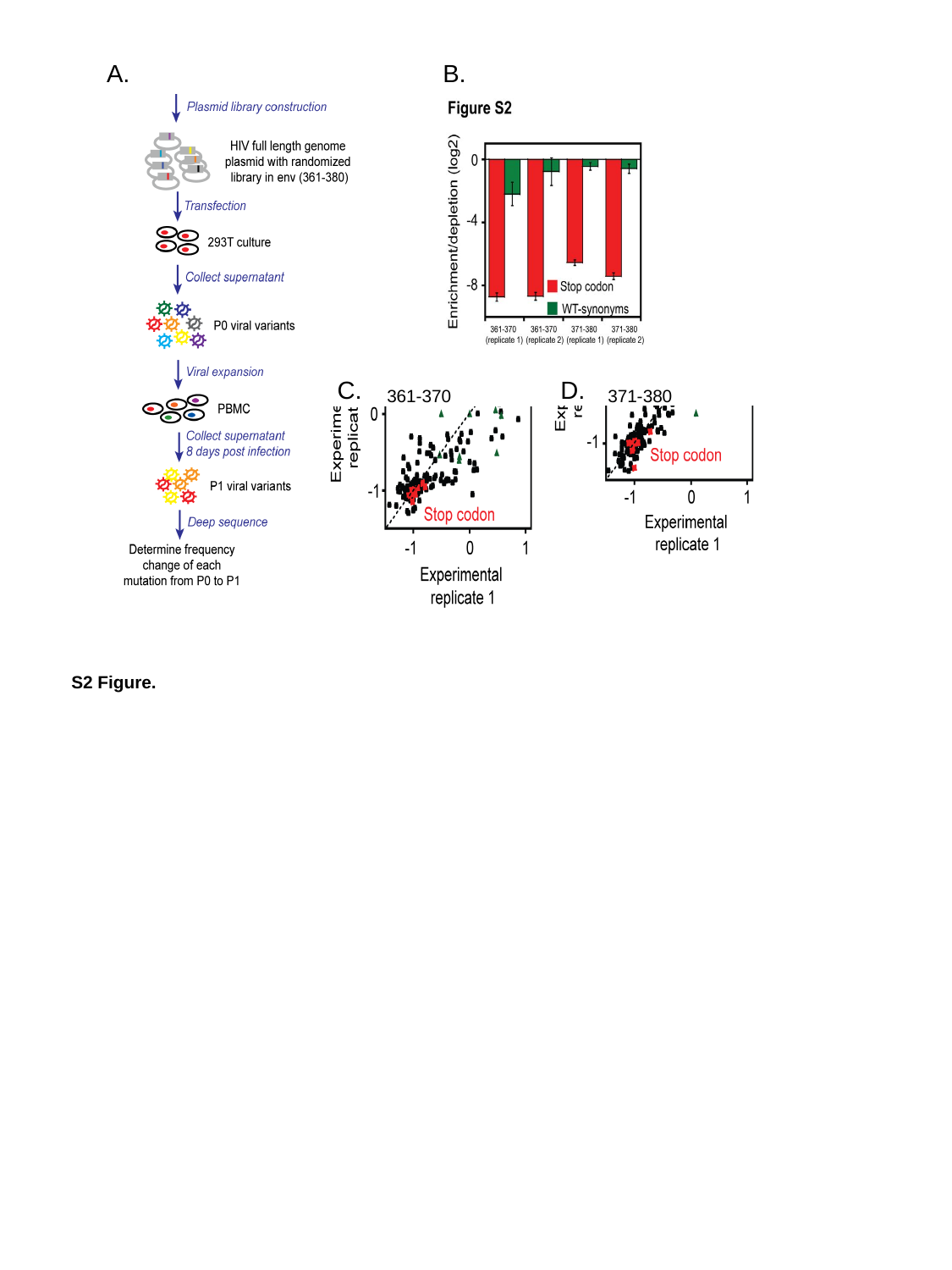

A.
B.
C.
D.
361-370
371-380
S2 Figure.

Supplement: S2 Fig — (A) A cassette ligation strategy was used to introduce all 64 possible codons into each position of the CD4 binding loop region to form two libraries encompassing residues 361–370 and 371–380. (B) Depletion of stop codons and enrichment of wt synonyms (change in log2 frequency) in two experimental replicates of each library. Stop codons are shown in red and wt synonyms are shown in green. (C and D) Reproducibility of EMPIRIC measurements in HIV. Correlation in selection coefficient of ~600 point mutants at amino acid positions 361–370 (C) and 371–380 (D) in the Env gene following infection of PBMCs. Green spots in panels B and C represent codons synonymous with wt codons. (PPTX) [file ppat.1005988.s016.pptx]

## Slide 1
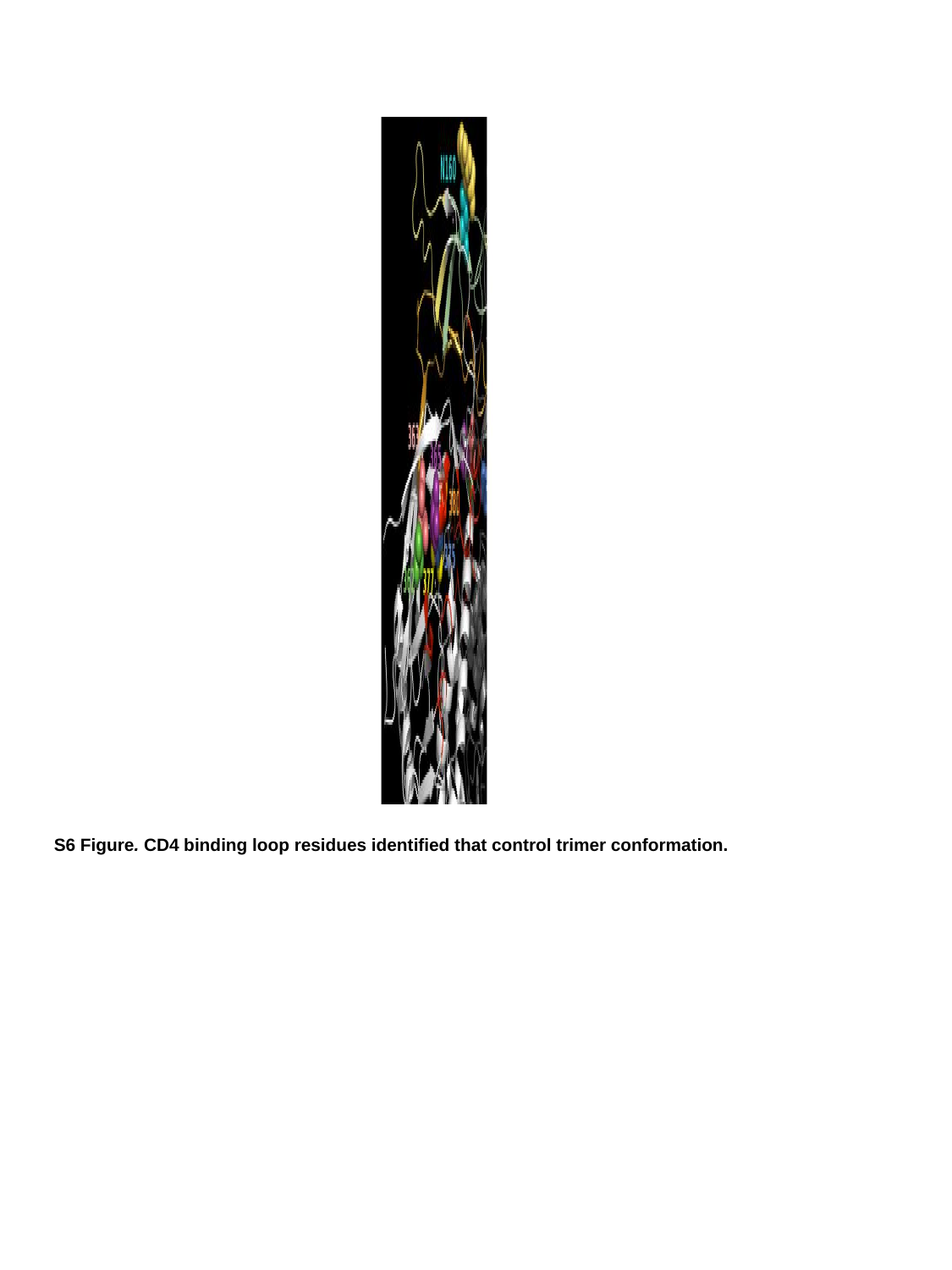

S6 Figure. CD4 binding loop residues identified that control trimer conformation.

Supplement: S6 Fig — Residues are depicted as spheres in different colors. The V1 (yellow), V2 (green) and V3 (orange) loops that form the trimer association domain (TAD) are shown at the trimer apex. Light yellow spheres show the NAG (N-acetyl glucosamine) of N160 (Cyan) on the top of the trimer. CD4 contact sites (2), are red in the cartoon structure. Structure is based on a side view of trimer (PDB 4NCO [12]). See also Movie at https://vimeo.com/165897330, password: Mama8). (PPTX) [file ppat.1005988.s020.pptx]
