# Supplementary material for: Saturation Mutagenesis of the HIV-1 Envelope CD4 Binding Loop Reveals Residues Controlling Distinct Trimer Conformations
Source: PLoS Pathog. 2016 Nov 7;12(11):e1005988. doi: 10.1371/journal.ppat.1005988 (PMC5098743; doi:10.1371/journal.ppat.1005988)
Supplement: S3 Fig — (A) ELISA showing enhanced exposure of the 17b epitope on LN40 monomeric gp120 in the presence of increasing concentrations of sCD4. (B) Introduction of N160 into LN40 did not confer sensitivity to neutralization by V2q mabs, PG9, PG16, PGT145. (C) PG9 and PGT145 do not bind monomeric Z1792M gp120. ELISAs showing HIV+ human serum mix (QC sera), PG9 and PGT145 interactions with captured gp120. (PPTX) [file ppat.1005988.s017.pptx]

## Slide 1
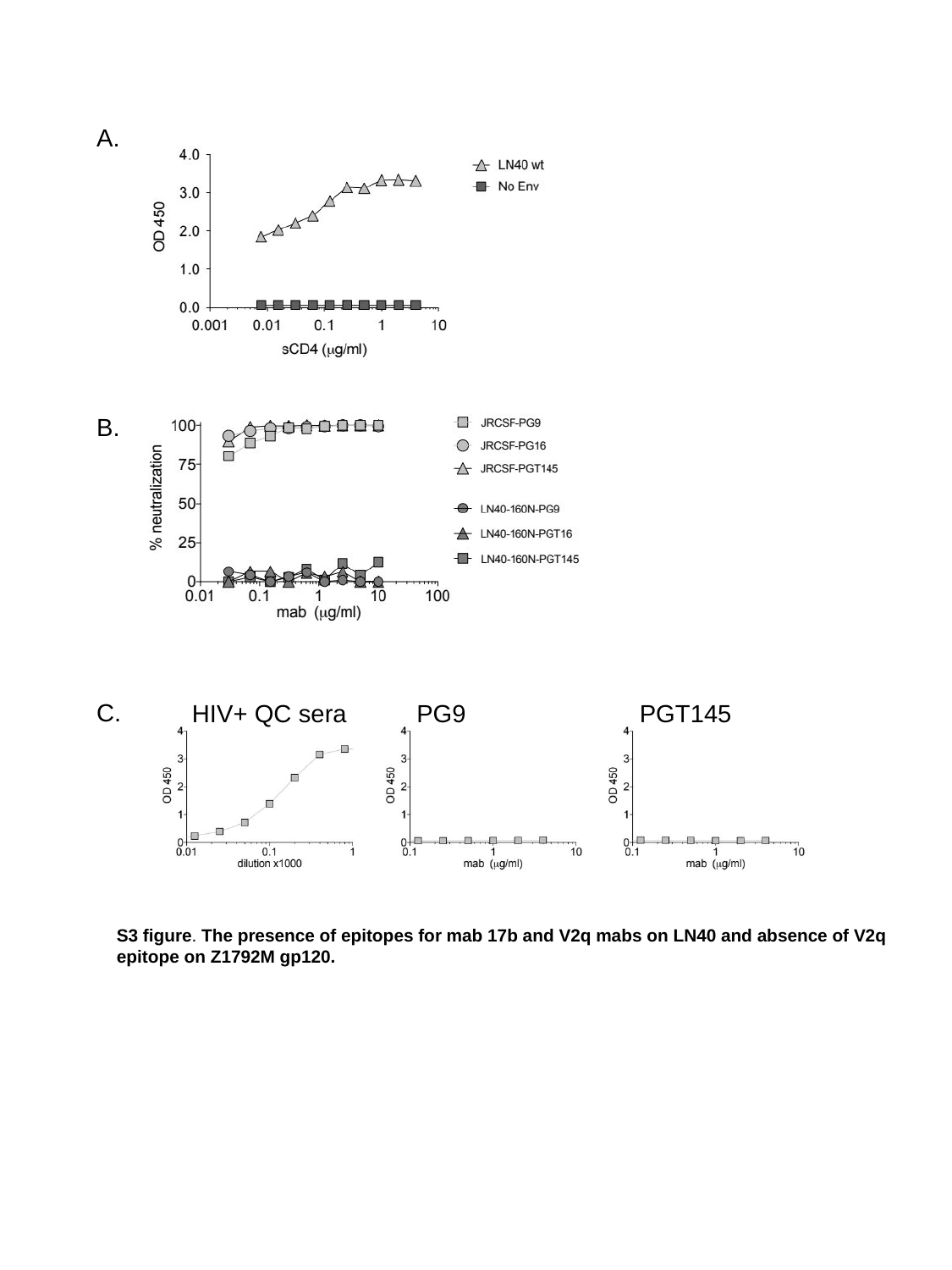

A.
B.
C.
HIV+ QC sera
PG9
PGT145
S3 figure. The presence of epitopes for mab 17b and V2q mabs on LN40 and absence of V2q epitope on Z1792M gp120.
