# Supplementary material for: Saturation Mutagenesis of the HIV-1 Envelope CD4 Binding Loop Reveals Residues Controlling Distinct Trimer Conformations
Source: PLoS Pathog. 2016 Nov 7;12(11):e1005988. doi: 10.1371/journal.ppat.1005988 (PMC5098743; doi:10.1371/journal.ppat.1005988)
Supplement: S4 Fig — LN8 375W Env was expressed on 293T cells, and stained with PGT145 mab in the presence and absence of sCD4. (PPTX) [file ppat.1005988.s018.pptx]

## Slide 1
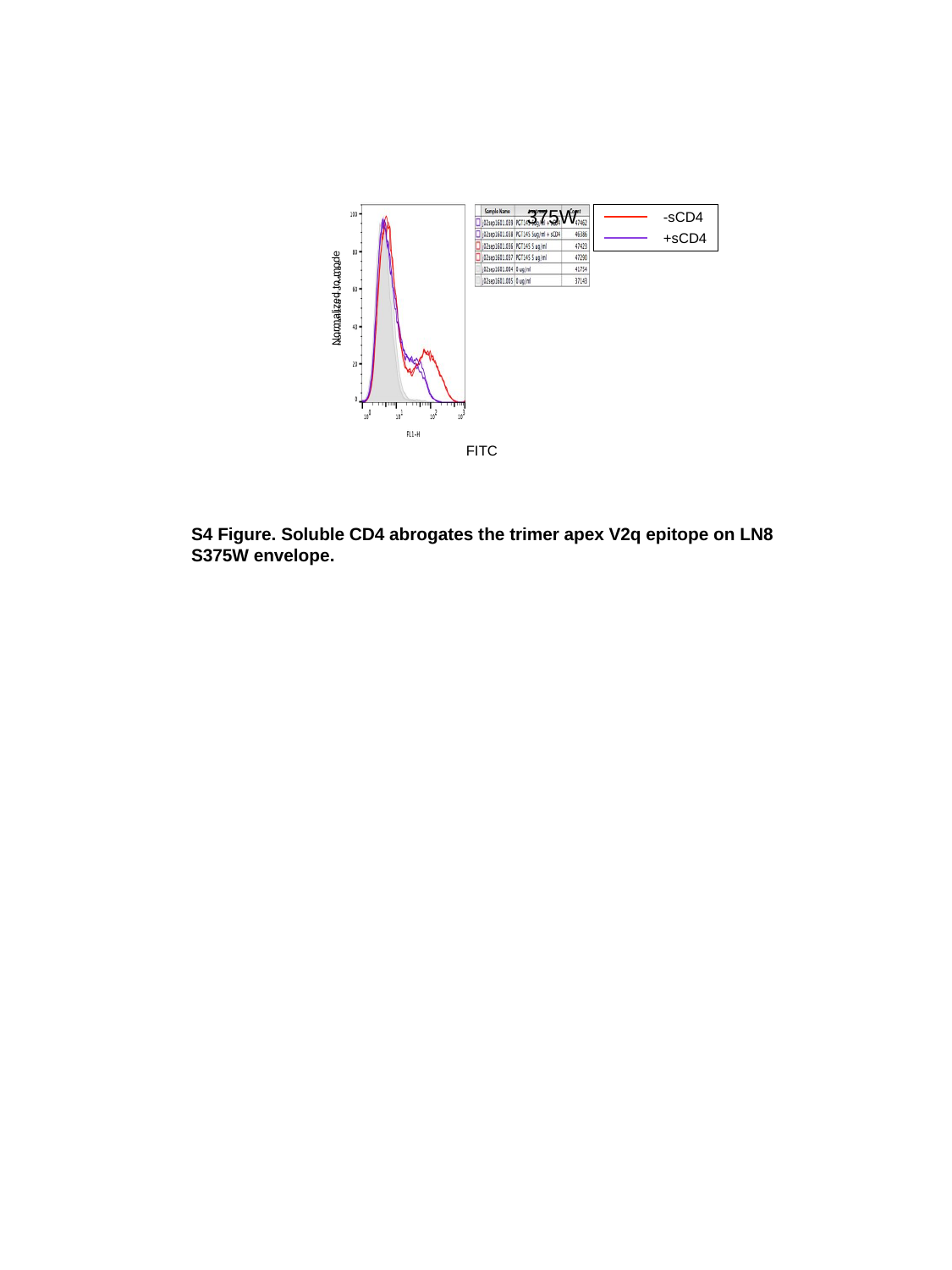

375W
-sCD4
+sCD4
Normalized to mode
FITC
S4 Figure. Soluble CD4 abrogates the trimer apex V2q epitope on LN8 S375W envelope.
