# Supplementary material for: Saturation Mutagenesis of the HIV-1 Envelope CD4 Binding Loop Reveals Residues Controlling Distinct Trimer Conformations
Source: PLoS Pathog. 2016 Nov 7;12(11):e1005988. doi: 10.1371/journal.ppat.1005988 (PMC5098743; doi:10.1371/journal.ppat.1005988)
Supplement: S5 Fig — (A) Localized conformational changes expose CD4bs epitopes without impacting the TAD and V2q epitopes. (B) The Env trimer moves from a closed to open form and back again. V2q mabs capture the closed form, CD4bs mabs capture the open form. (PPTX) [file ppat.1005988.s019.pptx]

## Slide 1
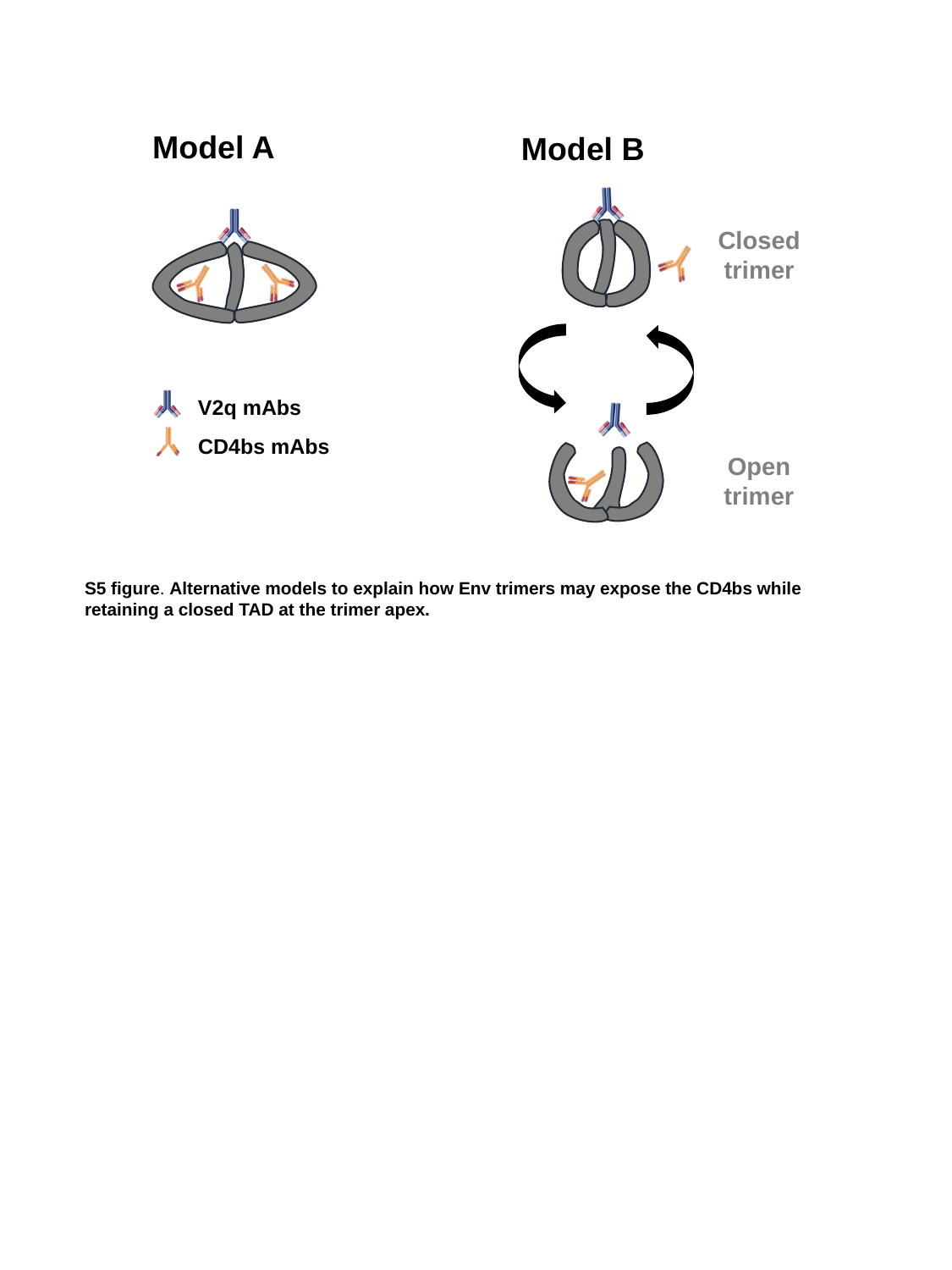

Model A
Model B
Closed trimer
Open trimer
V2q mAbs
CD4bs mAbs
S5 figure. Alternative models to explain how Env trimers may expose the CD4bs while retaining a closed TAD at the trimer apex.
